# Adverse events of antibody-drug conjugates: comparative analysis of agents with a common payload using the adverse event spontaneous reporting database

**DOI:** 10.1093/oncolo/oyaf298

**Published:** 2025-09-23

**Authors:** Kenta Yamaoka, Sho Masago, Mayako Uchida, Yoshihiro Uesawa, Nobuyuki Muroi, Tadashi Shimizu

**Affiliations:** Department of Pharmacy, Kobe City Medical Center General Hospital, Kobe, Hyogo, 650-0047, Japan; School of Pharmacy, Hyogo Medical University, Kobe, Hyogo, 650-8530, Japan; School of Pharmacy, Hyogo Medical University, Kobe, Hyogo, 650-8530, Japan; Department of Pharmacy, Shinko Hospital, Kobe, Hyogo, 651-0072, Japan; Department of Pharmacy, Kyushu University Hospital, Fukuoka, 812-8582, Japan; Department of Medical Molecular Informatics, Meiji Pharmaceutical University, Tokyo, 204-8588, Japan; Department of Pharmacy, Kobe City Medical Center General Hospital, Kobe, Hyogo, 650-0047, Japan; School of Pharmacy, Hyogo Medical University, Kobe, Hyogo, 650-8530, Japan

**Keywords:** antibody-drug conjugates, pharmacovigilance, polatuzumab vedotin, brentuximab vedotin, enfortumab vedotin

## Abstract

**Background:**

Antibody-drug conjugates (ADCs) have been implicated in myelosuppression and peripheral neuropathy (PN), possibly owing to their payload. Thus, their systemic toxicity should be considered. However, reports documenting adverse event (AE) occurrences in clinical settings are limited. This study analyzed AEs associated with polatuzumab vedotin, brentuximab vedotin, and enfortumab vedotin—which share a common payload—based on patient background factors using Japanese and United States AE spontaneous reporting databases.

**Materials and Methods:**

This study examined data from the Japanese Adverse Drug Event Report database (April 2004-June 2023) and the Food and Drug Administration AE Reporting System (first quarter of 1997-third quarter of 2023). Safety signals for an AEs were defined as a reporting odds ratio lower limit of the 95% confidence interval >1. Additionally, AEs were stratified based on sex, age, and body mass index to explore correlations between patient demographics and AEs.

**Results:**

Signals of PN, myelosuppression, and febrile neutropenia were detected in all ADCs. Polatuzumab vedotin showed earlier PN onset than other drugs and rare AEs such as heart failure. Enfortumab vedotin had widely detected skin-related AEs. Stratified analysis showed more AEs in males aged ≥60 years.

**Conclusions:**

Our study identifies common AEs, including PN and myelosuppression in ADCs sharing the same payload. Onset duration and outcomes of each AE vary among ADCs.

Implications for practice Antibody-drug conjugates (ADCs) carry payloads with strong anti-tumor effects due to their low-delivery rate to the target. Adverse effects due to the payload are severe, and peripheral neuropathy (PN) and other adverse events (AEs) have been reported in clinical trials. In this study, we analyzed ADCs with the same payload using the Japanese and US AE spontaneous reporting databases and detected common AE signals, including PN and myelosuppression. Additionally, cardiac dysfunction was observed with polatuzumab vedotin, underscoring the need for further investigation into concomitant medications and pharmacokinetics in the future.

## Introduction

Antibody-drug conjugates (ADCs) are pharmaceutical agents comprising a monoclonal antibody tailored to an antigen prominently expressed on neoplastic cells, alongside a cytotoxic drug payload, interconnected via a linker.^1–6^ ADCs selectively bind to target antigens and are internalized via pathways such as endocytosis.^1–3^ Thereafter, lysosomal degradation releases the payload and ultimately cellular demise.^2^^,^^3^^,^^7^ ADCs therapeutic potential hinges on their capacity to selectively transport a highly cytotoxic payload to the target site.^2^^,^^3^^,^^8^

Conventional monoclonal antibody drugs have a relatively high affinity for antigens expressed on the surface of cancer cells and have long half-lives; however, they can only target antigens expressed on the cell surface.^2^^,^^4^^,^^5^^,^^9^ Conversely, low molecular weight compounds used as payloads target intracellular proteins and other substances ^2^^,^^4^^,^^5^^,^^9^; additionally, they have low selectivity, frequently causing systemic toxicity. Thus, dose reduction or drug withdrawal is warranted. Combining these drugs allows selective tumor targeting with potent antitumor effects and the respective advantages of reduced off-target cytotoxicity.^1^^,^^2^^,^^4–6^

Approximately 2% of intravenously administered ADC payloads reach the target tumor cells.^10–14^ This is attributed to the delayed intracellular delivery of ADCs to tumor cells and the degradation of the linker and antibody before antigen binding.^1^^,^^4^ Cytotoxicity may occur in normal cells via non-specific engulfment and receptor-mediated endocytosis.^1^^,^^4^ Hence, payloads exhibiting mitotic inhibitory effects, less harmful to normal cells than neoplastic cells, are often preferred.^9^^,^^13^ Furthermore, payloads reaching neoplastic cells should exhibit antineoplastic effects at exceedingly low concentrations. Typically, payload drugs are 100-1000 times more potent than conventional cytotoxic agents used in clinical settings and are highly toxic for independent use.^3^^,^^9^^,^^13^

Monomethyl auristatin E (MMAE) serves as the payload for enfortumab vedotin, brentuximab vedotin, and polatuzumab vedotin among the ADCs currently approved in Japan.^15^ Analogous to vincristine, MMAE targets microtubules and induces cellular apoptosis by halting the cell cycle.^5^^,^^15^ As these ADCs use the same payload, similar adverse events (AEs) may be expressed. Peripheral neuropathy (PN) and myelosuppression, potentially caused by the payload, have been reported in clinical trials.^3^^,^^5^^,^^16–20^ Furthermore, all ADCs represent relatively novel therapeutic agents, and reports documenting AE occurrences in clinical settings are limited.^7^^,^^16–20^

Therefore, comparative analysis of AEs manifesting in ADCs sharing identical payloads is essential to understand differences in PN onset and other AEs. A comprehensive examination of these AEs can guide adverse drug reaction management and supportive care strategies in future clinical endeavors.

In this study, we examined AEs associated with polatuzumab vedotin, brentuximab vedotin, and enfortumab vedotin using the Japanese adverse drug event report (JADER) and the Food and Drug Administration adverse event reporting system (FAERS) databases. Furthermore, we compared the AE profiles of each drug with those of vincristine, a pharmaceutical agent sharing analogous payload effects, to elucidate disparities in AE occurrences among them. Additionally, the onset duration was assessed to understand the temporal manifestation of AEs associated with different monoclonal antibodies. Moreover, patients were stratified based on sex, age, and body mass index (BMI) to identify potential demographic-related discrepancies in AE occurrences.

## Materials and methods

### Data acquisition and preprocessing

The JADER dataset is accessible via the Pharmaceuticals and Medical Devices Agency website (https://www.pmda.go.jp). It comprises the following 4 distinct files: “demo,” “drug,” “reac,” and “hist.” The “demo” file includes fundamental patient characteristics such as sex, age, and reporting year, whereas the “drug” file contains details concerning medication (generic name), its trade name, administration route, date of initial administration, administration termination date, and drug involvement. “Reac” includes AE data (AE’s nomenclature, occurrence date, and outcome). “Hist” contains information regarding the patient’s underlying ailment. In each case, medications correlated with AEs were categorized into the following 3 groups: “suspect drugs,” “concomitant drugs,” and “interactions.” Cases classified as suspect drugs were extracted.

FAERS data spanning the first quarter of 1997 to the third quarter of 2023 were used in this study. The FAERS data structure adheres to the international safety reporting standards outlined in International Conference on Harmonization of Technical Requirements for Registration of Pharmaceuticals for Human Use Expert Working Group, including 7 distinct datasets: patient demographic and management information “DEMO,” drug/biologic details “DRUG,” AEs “REAC,” patient outcomes “OUTC,” reporting sources “RPSR,” drug therapy commencement and cessation dates “THER,” and indications for use/diagnosis “INDI.” They were analyzed and synthesized using CzeekV (https://www.czeek.com/), a platform configured and refined for integration with the aforementioned datasets.^21^^,^^22^ FAERS classifies the contribution of AEs involving the drug administered into 4 categories: “primary suspect drug,” “secondary suspect drug,” “concomitant,” and “interaction.” Cases classified as “primary suspect drugs” were extracted for analysis. The research protocol adhered to the Ministry of Health, Labour and Welfare’s Ethical Guidelines for Epidemiological Studies and the principles outlined in the Declaration of Helsinki. As an observational study utilizing anonymized data from the JADER and FAERS databases without involving any therapeutic interventions or human sample collection, the Ethical Review Committee of Hyogo Medical University determined that formal ethical approval was unnecessary. Furthermore, given that the study relied solely on publicly accessible data, obtaining individual patient consent was not required.

### Definition of AE names

Adverse event terms in the JADER and FAERS databases are based on preferred terms (PTs) from the Medical Dictionary for Regulatory Activities (MedDRA). In this study, AEs listed in PT were extracted from MedDRA version 26.0.

### Identification of AE signals

The analysis conducted using JADER included AEs with a frequency of ≥10 occurrences attributed to polatuzumab vedotin, brentuximab vedotin, and enfortumab vedotin.^23^^,^^24^ For FAERS, the top 50 AEs ranked by report frequency were analyzed. Thereafter, 2-by-2 contingency tables were created to calculate reporting odds ratios (RORs), structured by reports for the suspected drug, all other reports, reports with suspected AEs, and reports without suspected AEs (Table S1). The RORs used in pharmacovigilance for AE signal detection were calculated as (a × d)/(b × c) using the 2-by-2 contingency table. The criterion for detecting AE signals was met when the lower limit of the 95% confidence interval (CI) of the computed ROR was >1.

Additionally, to compare AEs based on the payload, disproportionalities were identified for the top 50 most reported AEs associated with vincristine, a drug sharing similar effects with MMAE, using JADER. Furthermore, post-expression outcomes for AEs demonstrating disproportionalities were analyzed.

### Creation of volcano plots

To visually interpret the relationship between each ADC and AEs, we created a scatter plot (volcano plot) comprising RORs and *P*-values. Volcano plots are frequently used in bioinformatics to identify trends in gene expression and AEs.^25^ This plot was created by transforming the ROR to the natural logarithm (ln (ROR)) on the *Y*-axis and the *P*-value of Fisher’s exact test to the ordinary logarithm [−log (*P*-value)] on the *X*-axis.^26^ The number of reported cases of each AE is indicated by a color, with the AE having the highest number of reported cases represented by a red dot. Thus, AEs plotted in the upper right quadrant marked with red dots visually represent disproportionalities due to the ADC of interest.

### Stratification analysis

ROR analysis for each ADC was conducted using JADER data, stratified based on age, sex, and BMI. Age groups, such as 50-59, were categorized as the 50th age group, and the analysis was performed using age 50 as the reference. Similarly, data pertaining to height and weight were processed and analyzed, and BMI was subsequently calculated based on these parameters.

Subsequently, age stratification involved classify the patient cohort into the following 2 categories: those aged <60 and ≥60 years. BMI analysis was categorized into 3 groups: <18.5kg/m^2^, 18.5–24.9 kg/m^2^, and ≥25 kg/m^2^.

Additionally, considering the reduction in case numbers due to stratified analyses and the potential difficulties in calculating the ROR, we employed an approximation method in this study by adding 0.5 to each cell value (a, b, c, and d) used for signal detection, as detailed in Table S1.^27^

Adverse events for which a signal was detected in each ADC were classified into categories including (1) hematological-related AEs, (2) infections, (3) tumor-related AEs, (4) neurologic-related AEs, (5) skin-related AEs, and (6) other.

### Analysis of the number of days to AE occurrence and the Weibull distribution

In this study, we analyzed the number of days until the AE onset, for which a signal was detected using JADER. To identify the timing of AE onset, the number of days to AE onset was calculated using the following formula, with data missing “date of start of administration” or “date of onset” excluded from the analysis:

Onset date of AE = “Date of onset of AE” − “Dose start date” + 0.5

For cases with multiple dosing start dates, the first dosing start date before the AE onset date was used in the calculation. In this study, the maximum time to AE onset considered for analysis was 2 years (730 days).^23^^,^^24^

The number of days to AE onset was fitted to a Weibull distribution on a cumulative incidence graph, which plotted failure rate instead of survival rate, using the calculated onset time for survival analysis. The Weibull distribution is represented by a scaling parameter (α) and a shape parameter (β), with the shape parameter used to evaluate the onset time profile of AEs. The shape parameter was used to evaluate the profile of the timing of AE onset among these parameters. When β = 1, the hazard was considered constant over time. When β was <1, the hazard was deemed to decrease over time (initial failure type). In contrast, when β was >1, the hazard was considered to increase over time (wear-out failure type). Statistical analysis was performed using JMP PRO15 (SAS Institute, Cary, NC, USA).

## Results

### AE signal analysis using the JADER database

From April 2004 to June 2023, JADER documented 2,200,140 cumulative AE records, with ROR analysis outcomes for each ADC summarized in Table 1. Polatuzumab vedotin was implicated as the suspect drug in 2,168 cases, with signal detection identified in 25 AEs. Brentuximab vedotin was implicated as the suspect drug in 2,673 cases, with signal detection in 19 AEs. Enfortumab vedotin was associated with 1688 reports, wherein 24 AEs exhibited signal detection. Vincristine appeared in 8,205 reports, with signals detected in 38 of the top 50 reported AEs (Table S2). Across all drugs, signals were detected for PN, myelosuppression, and febrile neutropenia (FN). Common signals among brentuximab vedotin, polatuzumab vedotin, and vincristine, used in hematopoietic tumors, included tumor lysis syndrome (TLS), *Pneumocystis jirovecii* pneumonia, and neutropenia. Conversely, enfortumab vedotin exhibited a higher incidence of skin-related AEs.

**Table 1.** Reports, ROR, and 95% CI for vedotin-containing ADCs associated with adverse events in JADER.

Polatuzumab vedotin			Brentuximab vedotin			Enfortumab vedotin		
**Variable**	Cases (*n*)	ROR (95% CI)	Variable	Cases (*n*)	ROR (95% CI)	Variable	Cases (*n*)	ROR (95% CI)
**CMV infection reactivation**	29	30.15 (20.79-43.72)	Anaplastic large-cell lymphoma	47	1356.19 (852.38-2157.79)	PN	264	58.98 (51.61-67.40)
**Lymphocyte count decreased**	116	28.79 (23.82-34.79)	Hodgkin’s disease recurrent	20	974.46 (509.89-1862.31)	Dermatitis bullous	12	46.84 (26.28-83.48)
**Cytopenia**	38	19.94 (14.42-27.56)	Peripheral T-cell lymphoma unspecified	30	134.81 (91.49-198.65)	Skin disorder	23	25.44 (16.79-38.54)
**CMV enterocolitis**	24	17.04 (11.36-25.56)	Hodgkin’s disease	90	130.63 (104.28-163.62)	Metastases to the liver	15	22.42 (13.43-37.43)
**CMV infection**	87	14.30 (11.52-17.75)	PN	403	57.62 (51.69-64.23)	Malignant neoplasm progression	77	15.32 (12.17-19.28)
**CRS**	13	13.23 (7.64-22.90)	Adult T-cell lymphoma/leukemia	12	46.30 (25.85-82.92)	Pulmonary toxicity	10	12.57 (6.73-23.47)
**Neutrophil count decreased**	348	13.22 (11.78-14.83)	Disease progression	138	37.98 (31.89-45.24)	Pyelonephritis	16	11.06 (6.75-18.14)
**CMV viremia**	27	12.66 (8.64-18.54)	Peripheral sensory neuropathy	10	17.82 (9.51-33.38)	Hyperglycemia	30	10.91 (7.59-15.68)
**TLS**	30	12.10 (8.42-17.39)	Condition aggravated	52	9.82 (7.45-12.95)	Myelosuppression	68	7.29 (5.71-9.29)
**Pneumonia cytomegaloviral**	10	10.83 (5.80-20.22)	Neutropenia	194	7.15 (6.17-8.27)	COVID-19	11	5.76 (3.18-10.44)
**White blood cell count decreased**	157	6.23 (5.30-7.33)	FN	188	6.99 (6.02-8.11)	TEN	23	3.51 (2.32-5.30)
**Platelet count decreased**	203	6.18 (5.35-7.15)	Neutrophil count decreased	202	5.62 (4.87-6.49)	Decreased appetite	42	3.38 (2.48-4.59)
**COVID-19**	15	6.13 (3.68-10.20)	Ileus paralytic	11	4.63 (2.56-8.38)	Pruritus	11	2.88 (1.59-5.21)
**Bacteremia**	10	5.80 (3.11-10.81)	TLS	10	3.21 (1.72-5.99)	SJS	23	2.35 (1.56-3.55)
**Anemia**	113	4.87 (4.03-5.89)	Myelosuppression	47	3.10 (2.32-4.14)	Interstitial lung disease	96	2.19 (1.78-2.70)
**FN**	106	4.73 (3.89-5.76)	*Pneumocystis jirovecii* pneumonia	28	2.81 (1.93-4.08)	Drug eruption	27	2.07 (1.41-3.03)
**Myelosuppression**	51	4.18 (3.16-5.51)	Pancreatitis acute	10	2.16 (1.16-4.02)	Erythema	14	2.05 (1.21-3.46)
**Neutropenia**	75	3.26 (2.59-4.10)	Sepsis	29	2.07 (1.44-2.99)	Rash	32	2.02 (1.42-2.86)
**Infection**	11	2.63 (1.45-4.76)	Pneumonia	67	1.94 (1.53-2.48)	FN	36	2.00 (1.44-2.79)
**PN**	16	2.28 (1.39-3.73)				Anemia	37	1.98 (1.43-2.74)
**Thrombocytopenia**	25	1.90 (1.28-2.82)				Erythema multiforme	17	1.90 (1.18-3.07)
**Sepsis**	21	1.85 (1.20-2.84)				Diarrhea	28	1.86 (1.28-2.70)
***Pneumocystis jirovecii* pneumonia**	15	1.85 (1.11-3.07)				Renal impairment	32	1.77 (1.25-2.51)
**Decreased appetite**	25	1.54 (1.04-2.29)				Pneumonia	36	1.65 (1.18-2.29)
**Pneumonia**	41	1.46 (1.07-1.98)						

Abbreviations: 95% CI, 95% confidence interval; CMV, cytomegalovirus; COVID-19, coronavirus disease 2019; CRS, cytokine release syndrome; FN, febrile neutropenia; PN, peripheral neuropathy; ROR, reported odds ratio; SJS, Stevens–Johnson syndrome; TEN, toxic epidermal necrolysis; TLS, tumor lysis syndrome

### AE signal analysis using the FAERS

FAERS recorded 17,272,063 aggregate reports from January 1997 to March 2023. Polatuzumab vedotin was implicated as the primary suspect drug in 3,848 reports. Brentuximab vedotin and enfortumab vedotin were reported in 7,936, and 1,761 cases, respectively. Analysis of the top 50 AEs reported for each drug revealed signal detection in 40, 36, and 27 AEs for enfortumab vedotin, brentuximab vedotin, and polatuzumab vedotin, respectively (Table 2). Signals common across all drugs included thrombocytopenia, neutropenia, disease progression, FN, PN, and anemia.

**Table 2.** Reports, ROR, and 95% CI for vedotin-containing ADCs associated with adverse events in FAERS.

Polatuzumab vedotin		Brentuximab vedotin		Enfortumab vedotin	
Variable	ROR (95% CI)	Variable	ROR (95% CI)	Variable	ROR (95% CI)
**CMV infection reactivation**	181.41 (145.86-225.64)	Anaplastic large-cell lymphoma	1427.40 (1100.08-1852.10)	Taste disorder	69.32 (48.27-99.56)
**DLBCL**	181.16 (157.95-207.79)	Hodgkin’s disease	410.80 (372.69-452.81)	Skin toxicity	53.00 (42.82-65.59)
**Lymphoma**	77.90 (68.68-88.37)	Peripheral sensory neuropathy	49.36 (40.59-60.02)	Hepatic function abnormal	48.30 (41.72-55.92)
**CMV test positive**	63.51 (44.48-90.68)	*Pneumocystis jirovecii* pneumonia	28.04 (23.50-33.44)	Myelosuppression	42.93 (34.21-53.86)
**Disease progression**	61.69 (57.64-66.03)	Polyneuropathy	27.07 (22.57-32.48)	TEN	29.81 (22.24-39.95)
**Blood lactate dehydrogenase**	57.05 (50.15-64.89)	FN	23.46 (21.46-25.65)	PN	28.44 (24.70-32.76)
**Hypogammaglobulinemia**	43.96 (32.82-58.87)	PN	16.86 (15.45-18.40)	Hyperglycemia	24.22 (19.71-29.77)
**CMV infection**	27.86 (22.84-33.99)	Neutrophil count decreased	9.90 (8.38-11.69)	Malignant neoplasm progression	21.97 (18.85-25.60)
**TLS**	25.00 (**1**8.56-33.67)	Neutropenia	9.48 (8.63-10.41)	SJS	20.89 (15.81-27.59)
**Neutrophil count decreased**	24.46 (21.16-28.28)	Pancytopenia	7.66 (6.58-8.93)	Skin disorder	20.17 (15.72-25.88)
**Lymphocyte count decreased**	23.96 (19.44-29.54)	Septic shock	7.66 (6.40-9.18)	Drug eruption	19.16 (13.43-27.33)
**Myelosuppression**	21.84 (17.75-26.86)	Disease progression	7.36 (6.56-8.25)	Metastases to the liver	14.43 (9.65-21.59)
**FN**	17.53 (15.32-20.05)	Thrombocytopenia	6.71 (5.99-7.53)	Neutrophil count decreased	10.71 (7.77-14.76)
**Neutropenia**	10.58 (9.39-11.91)	Leukopenia	5.89 (4.89-7.09)	Rash	7.59 (6.69-8.61)
**Pancytopenia**	10.31 (8.64-12.31)	Sepsis	5.59 (4.93-6.34)	Dry eye	7.35 (5.03-10.75)
**Cytopenia**	10.31 (8.64-12.31)	Infusion-related reaction	4.89 (4.04-5.91)	Interstitial lung disease	7.12 (4.99-10.15)
**COVID-19**	9.32 (8.25-10.52)	General physical health deterioration	4,84 (4,19-5.59)	FN	7.06 (5.18-9.62)
**Platelet count decreased**	9.11 (7.92-10.47)	Malignant neoplasm progression	4.59 (3.93-5.35)	Decreased appetite	6.50 (5.48-7.72)
**Thrombocytopenia**	8.90 (7.78-10.17)	White blood cell count decreased	4.41 (3.81-5.11)	Neutropenia	6.21 (4.95-7.80)
**C-reactive protein increased**	7.97 (6.23-10.21)	Respiratory failure	4.39 (3.68-5.23)	Alopecia	5.96 (4.93-7.22)
**White blood cell count decreased**	6,73 (5.74-7.90)	Pyrexia	4.29 (3.94-4.66)	Rash erythematous	5.41 (3.64-8.02)
**Aspartate aminotransferase increased**	6.71 (5.45-8.26)	Off-label use	4.01 (3.75-4.29)	Stomatitis	4.22 (2.82-6.32)
**PN**	6.32 (5.27-7.58)	Anemia	3.77 (3.35-4.24)	Skin exfoliation	4.14 (2.89-5.94)
**Anemia**	5.98 (5.27-6.79)	Pleural effusion	3.57 (2.90-4.39)	Disease progression	3.66 (2.65-5.04)
**Leukopenia**	5.84 (4.55-7.49)	Platelet count decreased	3.16 (2.66-3.76)	Thrombocytopenia	3.29 (2.39-4.54)
**Hepatic function abnormal**	4.78 (3.53-6.48)	Tachycardia	2.73 (2.26-3.31)	Renal impairment	3.27 (2.24-4.79)
**Therapeutic response decreased**	3.25 (2.38-4.44)	Infection	2,68 (2.27-3.17)	Anemia	3.22 (2.50-4.16)
		Weight decreased	2.46 (2.18-2.79)	Pruritus	3.04 (2.49-3.70)
		Hemoglobin decreased	2.44 (2.02-2.96)	Dehydration	3.02 (2.21-4.11)
		Hypotension	2.29 (1.98-2.65)	Sepsis	2.81 (1.98-3.99)
		Dehydration	2.26 (1.89-2.70)	Pyrexia	2.75 (2.24-3.37)
		Pneumonia	2.25 (2.00-2.53)	Acute kidney injury	2.74 (2.08-3.61)
		Chills	2.22 (1.84-2.69)	Inappropriate schedule of product administration	2.58 (1.95-3.40)
		Abdominal pain	2.10 (1.82-2.41)	Urinary tract infection	2.55 (1.87-3.49)
		Decreased appetite	1.80 (1.55-2.10)	Diarrhea	2.49 (2.10-2.95)
		Death	1.61 (1.47-1.76)	Malaise	2.39 (1.95-2.92)
				Hypoesthesia	2.33 (1.67-3.25)
				Weight decreased	2.22 (1.71-2.88)
				Fatigue	1.97 (1.65-2.34)
				Erythema	1.66 (1.18-2.35)

Abbreviations: 95% CI, 95% confidence interval; CMV, cytomegalovirus; COVID-19, coronavirus disease 2019; DLBCL, diffuse large B-cell lymphoma; FN, febrile neutropenia; PN, peripheral neuropathy; ROR, reported odds ratio; SJS, Stevens–Johnson syndrome; TEN, toxic epidermal necrolysis; TLS, tumor lysis syndrome.

### Volcano plot analysis

**Figure 1.**
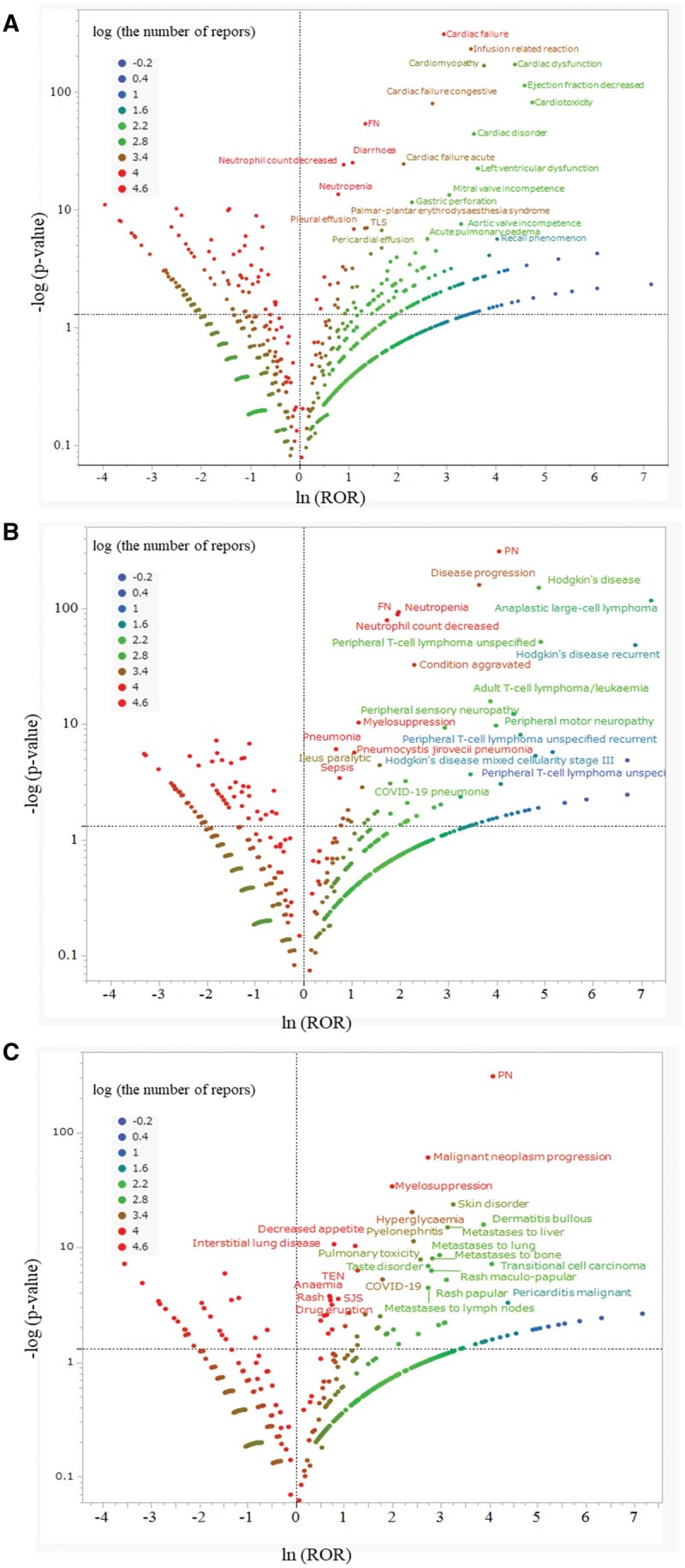
Volcano plot of AEs associated with ADCs. (A) Polatuzumab vedotin, (B) brentuximab vedotin, and (C) enfortumab vedotin. The *X*-axis shows the natural logarithms of the reported odds ratios (ln(ROR)), whereas the *Y*-axis represents the common logarithm of the inverse *P*-value (−log_10_(*P*-value)) derived from Fisher’s exact test. RORs were calculated using cross-tabulation. The dashed line indicates the signal detection threshold for lnROR [*x*-axis] >0 and −log_10_(*P*-value) [*y*-axis] >1.3. The red–green–blue points indicate the frequency of adverse effect reports (from −0.20 to 4.35). COVID-19, coronavirus disease 2019; FN, febrile neutropenia; PN, peripheral neuropathy; ROR, reported odds ratio; SJS, Stevens–Johnson syndrome; TEN, toxic epidermal necrolysis; TLS, tumor lysis syndrome.

Volcano plot analysis results for each medication are illustrated in Figure 1. Polatuzumab vedotin demonstrated a potential association with cardiotoxicity, encompassing heart failure and cardiomyopathy, alongside diarrhea, which eluded detection in the overall ROR analysis. Brentuximab vedotin and enfortumab vedotin demonstrated a strong association with PN, consistent with findings from the comprehensive ROR analysis.

### Stratified analysis using patient background

**Figure 2.**
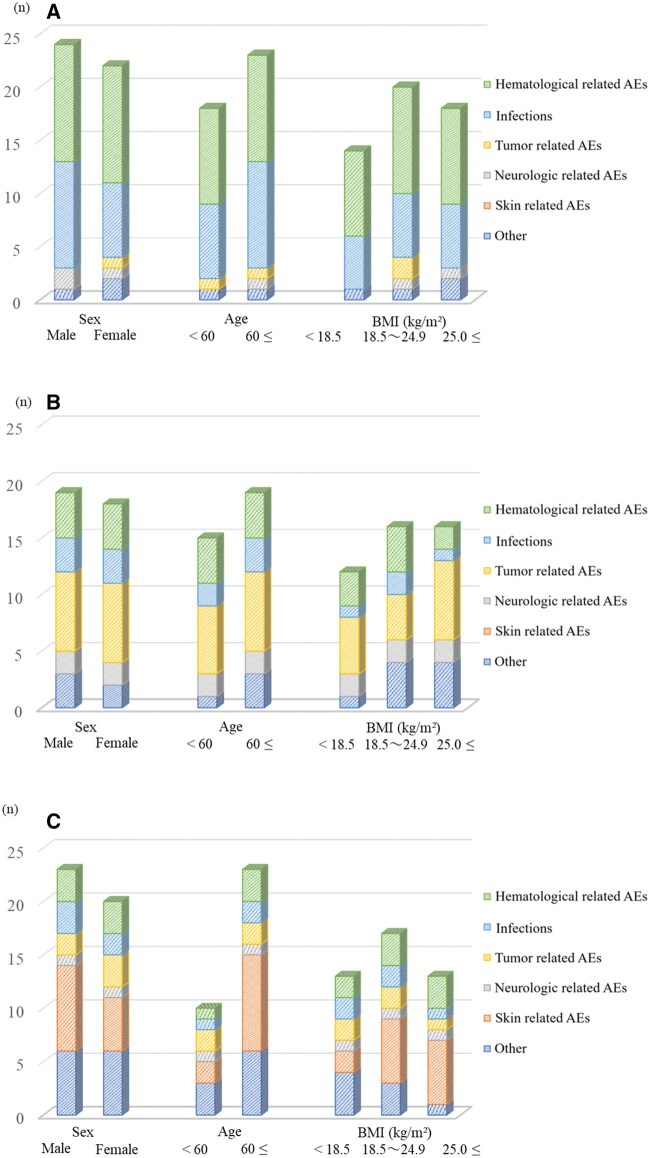
Distribution of the number of detected AE signals based on sex, age, and BMI strata. (A) Polatuzumab vedotin, (B) brentuximab vedotin, and (C) enfortumab vedotin. The number of adverse events with a lower limit of the ROR 95% CI >1 was plotted. Adverse events considered disproportionate were categorized into (1) Hematological AEs, (2) infections, (3) tumor-related AEs, (4) neurological-related AEs, (5) skin-related AEs, and (6) Others. Adverse events were categorized into the following groups. AE, adverse event; BMI, body mass index.

Figure 2 presents the analysis of AE signals using patient background factors, showing more AE signals in males and patients aged ≥60 years.

For polatuzumab vedotin, hematological AEs and infections, including cytomegalovirus were detected in both stratifications (Table S3). Additionally, signals not identified in the overall analysis, such as decreased hemoglobin in males and death and pancytopenia in females, were detected. No signals of PN and TLS were detected in patients with BMI <18.5 and ≤25.0 kg/m^2^, respectively.

For brentuximab vedotin, common signals were detected in AEs related to primary disease exacerbation, myelosuppression, and neurologic AEs including PN. New signals were detected in male patients with 18.5 kg/m^2^ ≤ BMI ≤ 24.9 kg/m^2^ and in interstitial lung disease in those with a BMI ≥25 kg/m^2^ (Table S4).

For enfortumab vedotin, skin-related and neurologic AEs were common signals detected in both the JADER and FAERS databases. Additionally, new signals were detected in death, fatigue, nausea, and neutropenia in females (Table S5).

### Outcomes after AEs

Following AEs with polatuzumab vedotin, 8 infection-related AEs, including sepsis (23.8%) and coronavirus disease 2019 (COVID-19) (13.3%), had a mortality rate exceeding 10% (Table S6).

Regarding brentuximab vedotin, all infection- and tumor-related AEs with detected signals exhibited a mortality percentage exceeding 10%, including TLS at 30.0%, which had a notably high mortality rate (Table S7).

In contrast to the other 2 medications, enfortumab vedotin depicted a lower mortality rate for infection-related AEs. Eight AEs, including toxic epidermal necrolysis (TEN) (43.5%) and malignant neoplasm progression (36.4%), reported a mortality rate exceeding 10%. Additionally, the percentage of individuals unrecovered from PN across all ADCs exceeded 30% (Table S8).

### Analysis of days to AE onset and Weibull distribution

**Figure 3.**
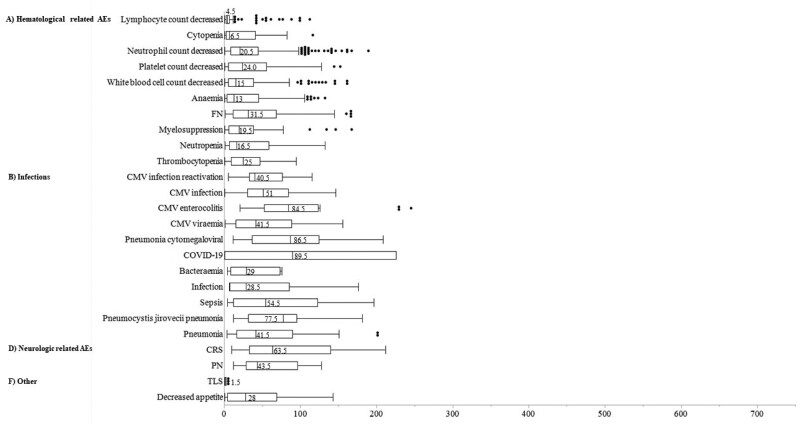
Box chart showing time to onset of adverse events associated with polatuzumab vedotin. Box plots show the 25th quartile, 75th quartile, and median (the vertical line inside the box). The whiskers reach the maximum and minimum values within 1.5 times the inner quartile range.

Figure 3 illustrates the time from treatment initiation to AE onset for polatuzumab vedotin. Most of the AEs were observed within 1 month from the start of treatment, with PN appearing within approximately 40 days.

**Figure 4.**
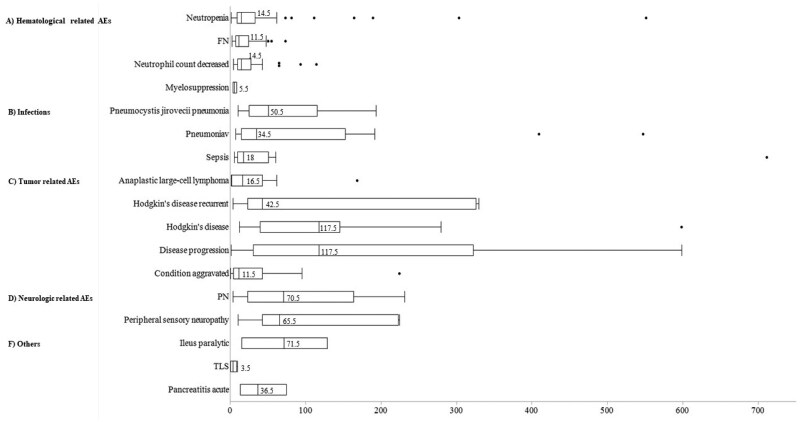
Box chart showing time to onset of adverse events associated with brentuximab vedotin. Box plots show the 25th quartile, 75th quartile, and median (the vertical line inside the box). The whiskers reach the maximum and minimum values within 1.5 times the inner quartile range.

For brentuximab vedotin, hematological AEs and infections typically occurred soon after treatment initiation, whereas neurologic AEs such as PN appeared after approximately 70 days (Figure 4).

**Figure 5.**
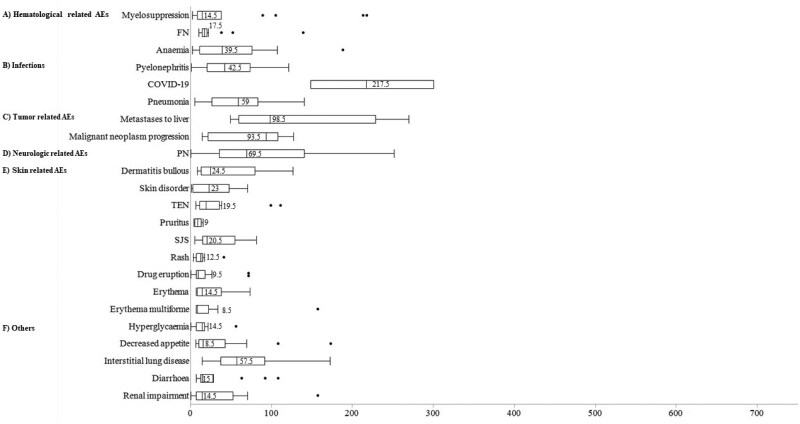
Box chart showing time to onset of adverse events associated with enfortumab vedotin. Box plots show the 25th quartile, 75th quartile, and median (the vertical line inside the box). The whiskers reach the maximum and minimum values within 1.5 times the inner quartile range.

For enfortumab vedotin, most skin-related AEs appeared in approximately 10 days (Figure 5). PN also emerged in approximately 70 days post-treatment initiation, as did brentuximab vedotin.

In the Weibull distribution analysis, early failure types (β > 1) included decreased lymphocyte count, cytopenia, platelet count, white blood cell count, and anemia for polatuzumab vedotin and sepsis for brentuximab vedotin (Tables S9 and S10). No early failure AEs were identified for enfortumab vedotin. Wear failure types, including COVID-19, pneumonia, pruritus, rash, and interstitial lung disease were observed for enfortumab vedotin (Table S11).

## Discussion

The advent of molecular targeted therapies has revolutionized cancer chemotherapy, with ADCs emerging as novel therapeutic entities alongside immune checkpoint inhibitors, garnering considerable attention in the medical realm. The 3 ADCs analyzed in this study share similar molecular weights, and the payload and linker attachment to individual monoclonal antibodies are presumed uniform.^28–30^ Although the carcinomas studied differ, drug doses are comparable; however, those dosing intervals for each vary: 3-week intervals for polatuzumab vedotin, 2–3 week intervals for brentuximab vedotin, and 3-week dosing with a 1-week rest for enfortumab vedotin.^16–18^ AE signals including PN, FN, and hematological issues, were identified in both analyses, potentially attributed to the payload MMAE, highlighting its potential role in systemic toxicity.

This study indicated that PN onset may manifest earlier with polatuzumab vedotin than with the other 2 agents, based on the analysis of days since treatment initiation. Although cumulative dosage is generally believed to influence PN onset, the lower exposure with polatuzumab vedotin, considering its dosing intervals, presents a paradoxical finding.^20^ A plausible explanation for these findings may be the influence of concurrent medications. Polatuzumab vedotin is usually administered alongside prophylactic azole antifungals because it is more susceptible to infection when combined with rituximab. Azole antifungal interacts via CYP3A4, potentially impacting MMAE metabolism. Although brentuximab vedotin and polatuzumab vedotin exhibit comparable PN incidence and severity, differences in concurrent medications influence outcomes.^31^ Furthermore, unlike ADCs, MMAE reaches peak blood concentrations 3 days post-administration, with concentrations remaining steady thereafter. This characteristic interaction with azole antifungal drugs may elevate concentrations, potentially leading to the early onset of AEs.^32^

In this study, polatuzumab vedotin was associated with cardiac-related AEs, including cardiac impairment, which were not identified in the overall ROR analysis; however, they were suggested in the volcano plot. Recently, the combination of R-CHP (rituximab, cyclophosphamide, doxorubicin, and prednisolone) has been reported as useful.^33^ Cardiac dysfunction is a typical AE of doxorubicin. As R-CHP was not approved in Japan until August 2022, the impact of this AE is currently minimal. It is anticipated that the frequency of cardiac dysfunction may increase in the future as R-CHP combination therapy becomes more widely used.

Adverse events reported in FAERS were generally similar to those reported in JADER; however, new AEs associated with hepatic dysfunction were identified. For brentuximab vedotin, signals detected included AEs related to disease exacerbation and paralytic ileus, with microtubule inhibitors generally causing such symptoms. PN-related AEs were detected in FAERS for brentuximab vedotin, along with less frequently reported AEs such as pleural effusion and tachycardia. Unlike the other 2 drugs, brentuximab vedotin uses chimeric antibodies as monoclonal antibodies, and infusion reaction signals were detected, possibly due to this effect.^4^

Brentuximab vedotin, similar to polatuzumab vedotin, is used in hematopoietic tumors; however, AEs such as heart failure were not detected in either analysis. This may be partly attributed to its use in combination with doxorubicin, cyclophosphamide, and prednisolone. AEs reported could be linked to doxorubicin use.^34^

Enfortumab vedotin showed a high number of skin-related AEs, possibly due to high Nectin-4 expression in skin tissues.^35^ Minor skin-related AEs are unlikely to be reported in JADER,^25^ whereas severe skin-related AEs, highlighted in clinical trials with enfortumab vedotin, likely increased reporting by healthcare providers.^18^^,^^36^^,^^37^ Severe skin-related AEs, such as Stevens–Johnson syndrome and TEN, were also reported, often appearing within the first month of treatment. Currently, limited information on prophylactic agents for skin-related AEs highlights the need for further research, emphasizing early treatment with antihistamines and steroids at symptom onset to reduce disease severity. Signals of renal dysfunction were also detected in both the JADER and FAERS databases.

Stratified analysis revealed more AE signals in patients aged ≥60 years across all drugs. This may be caused by age-related declines in organ function and a higher prevalence of diseases targeted by these drugs. Furthermore, AE signals were more prevalent in patients with a BMI of 18.5-24.9 kg/m^2^, possibly reflecting the smaller number of underweight and obese patients in the analyzed dataset.

This study had some limitations. First, only AEs that were experienced were reported in the JADER and FAERS databases, potentially introducing bias toward serious AEs or duplicate entries. Second, this study was limited by insufficient detailed information regarding concomitant medication regimens, dosage information, laboratory results, and patients’ prior treatment histories. Elevated MMAE blood levels in patients with hepatic dysfunction may have influenced AE reporting. Additionally, missing data on age, height, and weight prevented stratified analysis, leading to the exclusion of incomplete entries. Third, insufficient or missing data in the databases hindered the analysis of AE onset timing. Lastly, while our analysis highlights differences in AE profiles among these ADCs, multiple factors may influence these findings beyond the cytotoxic payload. Patient characteristics, disease type, target antigen distribution, differences in drug-to-antibody ratios, and variations in pharmacokinetics and potency can all contribute to observed AEs. Moreover, although toxicity is frequently attributed primarily to the payload, other structural components of ADCs—including the antibody and linker—may play a role in mediating adverse effects. These factors complicate attribution and should be considered when interpreting our results.

## Conclusion

Antibody-drug conjugates have developed globally since Paul Elrich proposed the “magic bullet” concept.^38^ This study analyzed AEs associated with 3 ADCs sharing the same payload, using JADER and FAERS data. Commonly identified AE signals included PN, myelosuppression, and FN, indicating their association with MMAE. Furthermore, polatuzumab vedotin showed trends toward cytomegalovirus-related infections and cardiac dysfunction, whereas enfortumab vedotin had skin-related AEs. Further research is needed to explore the interplay among AE occurrence, concomitant medications affecting ADC pharmacokinetics and MMAEs, and more detailed patient-specific characteristics using medical records, prescription databases, and other pertinent data sources. Importantly, PN and myelosuppression are well-recognized, mechanism-based AEs commonly associated with MMAE-containing ADCs. Our findings confirm these established toxicities using large, real-world pharmacovigilance datasets spanning 2 countries, while also demonstrating the utility of such cross-national analyses for uncovering additional or less-characterized safety signals that may vary based on patient populations, treatment practices, or ADC design features.

## Supplementary Material

oyaf298_Supplementary_Data

## Data Availability

The data that support the findings of this study are available at https://www.info.pmda.go.jp/fukusayoudb/CsvDownload.jsp and https://fis.fda.gov/extensions/FPD-QDE-FAERS/FPD-QDE-FAERS.html. All data generated or analyzed during this study are included in this article. Further enquiries can be directed to the corresponding author.
