# Supplementary material for: Adverse events of antibody-drug conjugates: comparative analysis of agents with a common payload using the adverse event spontaneous reporting database
Source: Oncologist. 2025 Sep 23;30(10):oyaf298. doi: 10.1093/oncolo/oyaf298 (PMC12517340; doi:10.1093/oncolo/oyaf298)
Supplement: oyaf298_Supplementary_Data [file oyaf298_supplementary_data.docx]

**Legends**

**Supplementary Table S1.** Two-by-two contingency table and calculation formula of ROR.

**Supplementary Table S2**. Reports, ROR, and 95% CI of vincristine-associated adverse events in JADER.

**Supplementary Table S3.** Stratified analysis of ROR and 95% CI of polatuzumab vedotin-associated adverse events in JADER

**Supplementary Table S4.** Stratified analysis of ROR and 95% CI of brentuximab vedotin-associated adverse events in JADER.

**Supplementary Table S5.** Stratified analysis of ROR and 95% CI of enfortumab vedotin-associated adverse events in JADER.

**Supplementary Table S6.** Proportion of post-event outcomes of polatuzumab vedotin-associated adverse events.

**Supplementary Table S7.** Proportion of post-event outcomes of brentuximab vedotin-associated adverse events.

**Supplementary Table S8.** Proportion of post-event outcomes of enfortumab vedotin-associated adverse events.

**Supplementary Table S9.** Weibull parameter of polatuzumab vedotin-associated adverse events.

**Supplementary Table S10.** Weibull parameter of brentuximab vedotin-associated adverse events.

**Supplementary Table S11.** Weibull parameter of enfortumab vedotin-associated adverse events.

**Supplementary Table S1.** Two-by-two contingency table and calculation formula of ROR.

|  | Reports with the suspected AE | Reports without the suspected AE |
| --- | --- | --- |
| Reports with the suspected drug | a | b |
| All other reports | c | d |

ROR (Reporting odds ratio) = (a/c) / (b/d) = a × d / b × c.

The cross-tabulation is structured into four categories: reports of suspected drugs, all other reports, the harmful event of interest, and the other harmful events. (a, b, c, and d indicate the respective number of cases in each category). The reporting odds ratio (ROR) was calculated as shown.

**Supplementary Table S2**. Reports, ROR, and 95% CI of vincristine-associated adverse events in JADER.

| Variable | Cases (n) | Non-cases (n) | Rate (%) | ROR (95% CI) |
| --- | --- | --- | --- | --- |
| Hepatitis B reactivation | 149 | 8056 | 1.82 | 17.44 (14.76-20.61) |
| MDS | 119 | 8086 | 1.45 | 9.46 (7.87-11.37) |
| Hepatitis C | 24 | 8181 | 0.29 | 8.64 (5.75-12.98) |
| CMV infection | 31 | 8174 | 0.38 | 8.43 (5.89-12.06) |
| Hepatitis B | 26 | 8179 | 0.32 | 8.38 (5.67-12.39) |
| CMV chorioretinitis | 30 | 8175 | 0.37 | 8.21 (5.71-11.82) |
| Posterior reversible encephalopathy syndrome | 50 | 8155 | 0.61 | 6.70 (5.06-8.88) |
| Myelosuppression | 294 | 7911 | 3.58 | 6.55 (5.82-7.37) |
| Small intestinal perforation | 26 | 8179 | 0.32 | 6.48 (4.39-9.57) |
| AML | 62 | 8143 | 0.76 | 6.30 (4.89-8.11) |
| White blood cell count decreased | 566 | 7639 | 6.90 | 5.99 (5.49-6.53) |
| Lymphocyte count decreased | 93 | 8112 | 1.13 | 5.79 (4.71-7.12) |
| TLS | 49 | 8156 | 0.60 | 5.21 (3.92-6.91) |
| Venoocclusive liver disease | 26 | 8179 | 0.32 | 4.72 (3.20-6.97) |
| Ileus paralytic | 33 | 8172 | 0.40 | 4.56 (3.23-6.44) |
| Leukopenia | 149 | 8056 | 1.82 | 4.34 (3.69-5.11) |
| Neutrophil count decreased | 468 | 7737 | 5.70 | 4.19 (3.81-4.60) |
| FN | 354 | 7851 | 4.31 | 4.19 (3.76-4.66) |
| Inappropriate antidiuretic hormone secretion | 49 | 8156 | 0.60 | 4.19 (3.15-5.55) |
| Bacteraemia | 25 | 8180 | 0.30 | 3.84 (2.59-5.71) |
| Neutropenia | 282 | 7923 | 3.44 | 3.25 (2.89-3.67) |
| Hepatic failure | 35 | 8170 | 0.43 | 3.05 (2.18-4.25) |
| Haemoglobin decreased | 110 | 8095 | 1.34 | 2.90 (2.40-3.51) |
| PN | 74 | 8131 | 0.90 | 2.81 (2.23-3.54) |
| Platelet count decreased | 342 | 7863 | 4.17 | 2.61 (2.34-2.90) |
| Infection | 38 | 8167 | 0.46 | 2.41 (1.75-3.32) |
| Haemophagocytic lymphohistiocytosis | 25 | 8180 | 0.30 | 2.33 (1.57-3.45) |
| Septic shock | 41 | 8164 | 0.50 | 2.30 (1.69-3.13) |
| Pancytopenia | 105 | 8100 | 1.28 | 2.18 (1.80-2.65) |
| Peritonitis | 27 | 8178 | 0.33 | 2.12 (1.45-3.10) |
| Thrombocytopenia | 104 | 8101 | 1.27 | 2.10 (1.73-2.54) |

**Supplementary Table S2**. Cont.

| Variable | Cases (n) | Non-cases (n) | Rate (%) | ROR (95% CI) |
| --- | --- | --- | --- | --- |
| Sepsis | 88 | 8117 | 1.07 | 2.05 (1.66-2.54) |
| Multiple organ dysfunction syndrome | 29 | 8176 | 0.35 | 1.90 (1.32-2.73) |
| Pneumocystis jirovecii pneumonia | 55 | 8150 | 0.67 | 1.79 (1.37-2.34) |
| Ileus | 24 | 8181 | 0.29 | 1.79 (1.20-2.67) |
| Anaemia | 156 | 8049 | 1.90 | 1.72 (1.46-2.01) |
| Alanine aminotransferase increased | 38 | 8167 | 0.46 | 1.52 (1.11-2.10) |
| Herpes zoster | 30 | 8175 | 0.37 | 1.48 (1.04-2.13) |

ROR, reported odds ratio; 95% CI, 95% confidence interval; MDS, myelodysplastic syndromes;CMV, cytomegalovirus; AML, acute myeloid leukemia; TLS, tumor lysis syndrome; FN, febrile neutropenia; PN, peripheral neuropathy.

**Supplementary Table S3.** Stratified analysis of ROR and 95% CI of polatuzumab vedotin-associated adverse events in JADER.

| Male | |  | Female | |  | Age < 60 | |  | 60 ≤ age | |
| --- | --- | --- | --- | --- | --- | --- | --- | --- | --- | --- |
| Variable | ROR (95% CI) |  | Variable | ROR (95% CI) |  | Variable | ROR (95% CI) |  | Variable | ROR (95% CI) |
| Lymphocyte count decreased | 29.00  (22.21-37.86) |  | CMV infection reactivation | 41.45  (24.86-69.09) |  | Cytopenia | 47.84  (24.03-95.26) |  | CMV infection reactivation | 33.28  (22.13-50.07) |
| CMV infection reactivation | 24.44  (14.46-41.29) |  | Lymphocyte count decreased | 32.69  (25.01-42.74) |  | CRS | 39.96  (17.08-93.50) |  | Lymphocyte count decreased | 30.29  (24.70-37.15) |
| Cytopenia | 23.96  (15.98-35.92) |  | CMV enterocolitis | 21.65  (12.04-38.95) |  | CMV infection reactivation | 38.28  (16.37-89.56) |  | CMV infection | 24.25  (19.33-30.44) |
| Pneumonia cytomegaloviral | 16.22  (8.22-32.04) |  | TLS | 19.84  (12.14-32.42) |  | Lymphocyte count decreased | 30.12  (17.98-50.48) |  | CMV viraemia | 23.90  (15.93-35.86) |
| CMV enterocolitis | 15.22  (8.86-26.14) |  | Neutrophil count decreased | 16.93  (14.39-19.92) |  | TLS | 20.31 (7.97-51.76) |  | CMV enterocolitis | 18.62  (12.22-28.38) |
| CMV infection | 14.46  (10.85-19.26) |  | Cytopenia | 15.90 (8.85-28.56) |  | Neutrophil count decreased | 15.12  (10.68-21.41) |  | Cytopenia | 16.70  (11.41-24.44) |
| CRS | 14.08 (7.13-27.78) |  | CMV viraemia | 15.31 (8.92-26.30) |  | CMV enterocolitis | 13.07 (3.75-45.55) |  | Pneumonia cytomegaloviral | 11.94 (6.47-22.01) |
| CMV viraemia | 11.92 (7.08-20.07) |  | CRS | 15.24 (6.55-35.44) |  | COVID-19 | 11.44 (3.98-32.93) |  | Neutrophil count decreased | 11.79  (10.43-13.33) |
| Neutrophil count decreased | 10.87 (9.18-12.86) |  | CMV infection | 13.51 (9.55-19.11) |  | Bacteraemia | 11.21 (3.22-39.05) |  | CRS | 10.56 (5.36-20.83) |
| Bacteraemia | 7.84 (3.81-16.12) |  | White blood cell count decreased | 7.54 (6.01-9.45) |  | Platelet count decreased | 11.06 (7.53-16.26) |  | TLS | 9.86 (6.65-14.62) |
| TLS | 7.59 (4.34-13.29) |  | Platelet count decreased | 7.53 (6.12-9.26) |  | White blood cell count decreased | 9.91 (6.62-14.83) |  | COVID-19 | 7.18 (4.11-12.56) |
| COVID-19 | 7.39 (4.01-13.60) |  | Pneumonia cytomegaloviral | 6.38 (1.84-22.15) |  | Death | 8.42 (3.86-18.37) |  | Bacteraemia | 5.84 (2.97-11.49) |
| White blood cell count decreased | 5.46 (4.31-6.92) |  | FN | 5.37 (3.95-7.28) |  | CMV viraemia | 8.38 (2.91-24.10) |  | White blood cell count decreased | 5.58 (4.67-6.67) |
| Platelet count decreased | 5.12 (4.15-6.31) |  | Anaemia | 4.96 (3.77-6.52) |  | Anaemia | 7.58 (4.53-12.68) |  | Platelet count decreased | 5.02 (4.30-5.87) |
| Anaemia | 5.07 (3.91-6.59) |  | Myelosuppression | 4.83 (3.23-7.23) |  | Haemoglobin decreased | 7.45 (3.42-16.26) |  | Myelosuppression | 4.32 (3.23-5.78) |
| FN | 4.31 (3.31-5.61) |  | Bacteraemia | 4.81 (1.68-13.77) |  | FN | 4.71 (2.54-8.75) |  | FN | 4.23 (3.42-5.23) |

**Supplementary Table S3.** Cont.

| Male | |  | Female | |  | Age < 60 | |  | 60 ≤ age | |
| --- | --- | --- | --- | --- | --- | --- | --- | --- | --- | --- |
| Variable | ROR (95% CI) |  | Variable | ROR (95% CI) |  | Variable | ROR (95% CI) |  | Variable | ROR (95% CI) |
| Infection | 4.06 (2.14-7.70) |  | COVID-19 | 3.76 (1.31-10.75) |  | Thrombocytopenia | 3.79 (1.49-9.64) |  | Anaemia | 4.09 (3.34-5.01) |
| Myelosuppression | 3.59 (2.38-5.40) |  | Neutropenia | 3.28 (2.28-4.70) |  | Myelosuppression | 3.44 (1.35-8.74) |  | Neutropenia | 3.63 (2.86-4.60) |
| Neutropenia | 3.50 (2.57-4.78) |  | Death | 2.80 (1.52-5.15) |  |  |  |  | Infection | 2.83 (1.54-5.19) |
| PN | 3.23 (1.85-5.65) |  | Decreased appetite | 2.40 (1.45-3.97) |  |  |  |  | PN | 2.58 (1.56-4.25) |
| Pneumocystis jirovecii pneumonia | 2.75 (1.49-5.05) |  | Thrombocytopenia | 2.15 (1.20-3.85) |  |  |  |  | Sepsis | 1.91 (1.24-2.96) |
| Haemoglobin decreased | 2.00 (1.09-3.67) |  | Pancytopenia | 2.12 (1.21-3.70) |  |  |  |  | Pneumocystis jirovecii pneumonia | 1.88 (1.14-3.10) |
| Sepsis | 1.98 (1.16-3.40) |  |  |  |  |  |  |  | Thrombocytopenia | 1.61 (1.05-2.47) |
| Thrombocytopenia | 1.97 (1.17-3.30) |  |  |  |  |  |  |  |  |  |

**Supplementary Table S3.** Cont.

| BMI < 18.5 kg/m2 | |  | 18.5 kg/m^2^ ≤ BMI ≤ 24.9 kg/m^2^ | |  | BMI ≤ 25.0 kg/m^2^ | |
| --- | --- | --- | --- | --- | --- | --- | --- |
| Variable | ROR (95% CI) |  | Variable | ROR (95% CI) |  | Variable | ROR (95% CI) |
| CMV infection reactivation | 66.91 (36.76-121.78) |  | CMV infection reactivation | 91.06 (52.39-158.26) |  | Pneumonia cytomegaloviral | 71.21 (29.42-172.36) |
| Lymphocyte count decreased | 15.13 (10.31-22.20) |  | Lymphocyte count decreased | 22.06 (17.07-28.50) |  | CMV viraemia | 70.07 (28.97-169.50) |
| Neutrophil count decreased | 14.03 (11.26-17.49) |  | CMV enterocolitis | 21.38 (12.56-36.37) |  | Cytopenia | 45.40 (17.37-118.65) |
| TLS | 13.50 (6.79-26.86) |  | Cytopenia | 18.75 (9.76-36.02) |  | CMV enterocolitis | 36.63 (10.22-131.23) |
| CMV viraemia | 10.28 (4.70-22.48) |  | CMV viraemia | 15.14 (8.58-26.71) |  | CMV infection | 23.56 (12.16-45.62) |
| Bacteraemia | 9.59 (3.76-24.50) |  | CMV infection | 12.20 (8.47-17.59) |  | PN | 16.78 (7.62-36.98) |
| CMV infection | 9.44 (5.23-17.04) |  | Neutrophil count decreased | 10.66 (9.03-12.59) |  | Lymphocyte count decreased | 13.67 (6.55-28.53) |
| CMV enterocolitis | 8.64 (3.39-22.05) |  | TLS | 7.79 (4.22-14.39) |  | Infection | 12.05 (3.43-42.36) |
| Anaemia | 6.91 (5.00-9.55) |  | CRS | 6.22 (1.78-21.73) |  | Neutrophil count decreased | 9.45 (6.26-14.27) |
| Platelet count decreased | 6.35 (4.85-8.30) |  | Bacteraemia | 5.55 (2.19-14.10) |  | White blood cell count decreased | 7.50 (4.74-11.88) |
| Myelosuppression | 5.43 (3.36-8.77) |  | Anaemia | 5.26 (4.04-6.85) |  | Anaemia | 5.73 (3.07-10.70) |
| White blood cell count decreased | 4.92 (3.55-6.83) |  | Platelet count decreased | 5.04 (4.09-6.21) |  | Neutropenia | 5.35 (2.78-10.29) |
| FN | 3.81 (2.60-5.56) |  | White blood cell count decreased | 5.01 (3.99-6.28) |  | FN | 4.88 (2.61-9.10) |
| Neutropenia | 3.08 (1.88-5.04) |  | Death | 3.89 (2.11-7.16) |  | Myelosuppression | 4.45 (1.74-11.37) |
|  |  |  | FN | 3.15 (2.33-4.26) |  | Platelet count decreased | 4.41 (2.58-7.54) |
|  |  |  | Neutropenia | 2.82 (2.00-3.99) |  | Decreased appetite | 3.44 (1.35-8.78) |
|  |  |  | Myelosuppression | 2.58 (1.56-4.26) |  | Thrombocytopenia | 3.00 (1.04-8.66) |
|  |  |  | PN | 2.54 (1.17-5.52) |  | Pyrexia | 2.68 (1.22-5.86) |
|  |  |  | Sepsis | 2.24 (1.31-3.84) |  |  |  |
|  |  |  | Thrombocytopenia | 1.92 (1.14-3.22) |  |  |  |

ROR, reported odds ratio; 95% CI, 95% confidence interval; CMV, cytomegalovirus; CRS, cytokine release syndrome; TLS, tumor lysis syndrome; COVID-19, Coronavirus disease 2019; FN, febrile neutropenia; PN, peripheral neuropathy.

| Male | |  | Female | |  | Age < 60 | |  | 60 ≤ age | |
| --- | --- | --- | --- | --- | --- | --- | --- | --- | --- | --- |
| Variable | ROR (95% CI) |  | Variable | ROR (95% CI) |  | Variable | ROR (95% CI) |  | Variable | ROR (95% CI) |
| Hodgkin's disease recurrent | 618.57  (267.57-1430.04) |  | Anaplastic large-cell lymphoma | 50054.18  (3013.65-831357.92) |  | Anaplastic large-cell lymphoma | 1641.50  (684.69-3935.40) |  | Anaplastic large-cell lymphoma | 1121.36  (653.11-1925.35) |
| Anaplastic large-cell lymphoma | 614.72  (362.95-1041.12) |  | Hodgkin's disease recurrent | 1538.96  (537.10-4409.64) |  | Hodgkin's disease recurrent | 786.48  (154.92-3992.65) |  | Hodgkin's disease recurrent | 835.37  (415.84-1678.16) |
| Hodgkin's disease | 148.97  (109.48-202.70) |  | Peripheral T-cell lymphoma unspecified | 206.93  (110.77-386.57) |  | Hodgkin's disease | 169.85  (113.02-255.24) |  | Peripheral T-cell lymphoma unspecified | 169.65  (109.76-262.21) |
| Peripheral T-cell lymphoma unspecified | 99.84  (58.45-170.54) |  | Hodgkin's disease | 116.68  (78.54-173.34) |  | PN | 72.84 (60.18-88.17) |  | Hodgkin's disease | 113.19  (85.75-149.42) |
| PN | 48.19 (41.56-55.87) |  | Adult T-cell lymphoma/leukaemia | 101.21  (52.14-196.46) |  | Peripheral T-cell lymphoma unspecified | 64.94 (25.03-168.50) |  | PN | 47.38 (41.08-54.65) |
| Disease progression | 31.26 (24.73-39.52) |  | PN | 58.68 (48.01-71.71) |  | Disease progression | 40.34 (27.90-58.31) |  | Disease progression | 37.44 (30.41-46.10) |
| Adult T-cell lymphoma/leukaemia | 27.94 (9.58-81.47) |  | Disease progression | 49.75 (36.70-67.45) |  | Peripheral sensory neuropathy | 18.79 (5.37-65.72) |  | Adult T-cell lymphoma/leukaemia | 34.99 (16.78-72.99) |
| Peripheral sensory neuropathy | 17.73 (7.59-41.40) |  | Peripheral sensory neuropathy | 31.74 (13.58-74.15) |  | Neutropenia | 11.49 (9.05-14.59) |  | Peripheral sensory neuropathy | 19.87 (10.03-39.36) |
| Condition aggravated | 11.16 (7.90-15.76) |  | Condition aggravated | 9.67 (5.84-16.01) |  | Neutrophil count decreased | 8.21 (6.36-10.61) |  | Condition aggravated | 11.97 (8.81-16.27) |
| Neutropenia | 7.50 (6.21-9.06) |  | Neutropenia | 7.71 (5.89-10.10) |  | FN | 8.09 (6.09-10.75) |  | Ileus paralytic | 7.22 (3.81-13.71) |
| FN | 6.86 (5.71-8.24) |  | Neutrophil count decreased | 7.08 (5.55-9.01) |  | Condition aggravated | 7.86 (4.27-14.49) |  | FN | 6.41 (5.35-7.69) |
| Ileus paralytic | 5.77 (3.04-10.95) |  | FN | 6.15 (4.50-8.42) |  | TLS | 5.26 (1.84-15.08) |  | Neutropenia | 5.93 (4.87-7.21) |
| Neutrophil count decreased | 5.25 (4.33-6.35) |  | TLS | 4.89 (1.71-14.01) |  | Pneumocystis jirovecii pneumonia | 4.21 (2.22-7.99) |  | Neutrophil count decreased | 4.82 (4.04-5.75) |
| Pneumocystis jirovecii pneumonia | 3.51 (2.22-5.56) |  | Pancreatitis acute | 4.78 (2.06-11.07) |  | Myelosuppression | 2.97 (1.66-5.32) |  | Myelosuppression | 3.22 (2.29-4.54) |

**Supplementary Table S4.** Stratified analysis of ROR and 95% CI of brentuximab vedotin-associated adverse events in JADER.

| Male | |  | Female | |  | Age < 60 | |  | 60 ≤ age | |
| --- | --- | --- | --- | --- | --- | --- | --- | --- | --- | --- |
| Variable | ROR (95% CI) |  | Variable | ROR (95% CI) |  | Variable | ROR (95% CI) |  | Variable | ROR (95% CI) |
| TLS | 2.82 (1.30-6.11) |  | Myelosuppression | 4.10 (2.55-6.59) |  | Pneumonia | 1.92 (1.07-3.45) |  | TLS | 2.97 (1.45-6.09) |
| Myelosuppression | 2.69 (1.80-4.01) |  | Pneumocystis jirovecii pneumonia | 2.86 (1.51-5.43) |  |  |  |  | Pancreatitis acute | 2.85 (1.32-6.17) |
| Fall | 2.27 (1.16-4.46) |  | Sepsis | 2.60 (1.37-4.94) |  |  |  |  | Sepsis | 2.39 (1.61-3.57) |
| Sepsis | 2.07 (1.33-3.24) |  | Pneumonia | 1.92 (1.18-3.12) |  |  |  |  | Pneumocystis jirovecii pneumonia | 2.35 (1.49-3.72) |
| Pneumonia | 1.88 (1.40-2.54) |  |  |  |  |  |  |  | Pneumonia | 1.92 (1.47-2.51) |

**Supplementary Table S4.** Cont.

**Supplementary Table S4.** Cont.

| BMI < 18.5 kg/m^2^ | |  | 18.5 kg/m^2^ ≤ BMI ≤ 24.9 kg/m^2^ | |  | BMI ≤ 25.0 kg/m^2^ | |
| --- | --- | --- | --- | --- | --- | --- | --- |
| Variable | ROR (95% CI) |  | Variable | ROR (95% CI) |  | Variable | ROR (95% CI) |
| Anaplastic large-cell lymphoma | 7045.86  (336.94-147337.80) |  | Hodgkin's disease recurrent | 3564.80  (144.96-87663.69) |  | Anaplastic large-cell lymphoma | 3207.30  (130.09-79072.74) |
| Hodgkin's disease recurrent | 4201.81  (170.54-103526.93) |  | Anaplastic large-cell lymphoma | 557.73  (156.13-1992.33) |  | Hodgkin's disease recurrent | 3207.30  (130.09-79072.74) |
| Peripheral T-cell lymphoma unspecified | 280.11  (48.20-1627.70) |  | Hodgkin's disease | 83.61 (31.97-218.70) |  | Adult T-cell lymphoma/leukaemia | 1061.54  (20.99-53687.99) |
| Hodgkin's disease | 115.49  (31.52-423.14) |  | Peripheral sensory neuropathy | 58.25 (26.32-128.89) |  | Peripheral T-cell lymphoma unspecified | 246.71  (41.48-1467.47) |
| Peripheral sensory neuropathy | 40.01 (7.84-204.24) |  | Condition aggravated | 20.29 (10.21-40.29) |  | Hodgkin's disease | 128.28  (23.40-703.40) |
| Condition aggravated | 18.18 (7.09-46.67) |  | Ileus paralytic | 15.55 (6.10-39.66) |  | Peripheral sensory neuropathy | 101.56  (27.48-375.42) |
| PN | 13.76 (5.37-35.27) |  | PN | 10.90 (5.70-20.83) |  | Disease progression | 21.78 (6.16-77.02) |
| FN | 6.44 (3.82-10.88) |  | Neutrophil count decreased | 7.44 (5.43-10.20) |  | TLS | 15.41 (4.37-54.29) |
| Pancreatitis acute | 6.19 (1.77-21.65) |  | TLS | 7.34 (2.55-21.09) |  | Condition aggravated | 11.56 (3.29-40.64) |
| Pneumocystis jirovecii pneumonia | 5.63 (1.95-16.28) |  | FN | 6.69 (4.64-9.66) |  | FN | 10.48 (6.17-17.79) |
| Neutrophil count decreased | 5.54 (3.28-9.35) |  | Fall | 5.73 (2.26-14.55) |  | PN | 8.57 (2.44-30.07) |
| Neutropenia | 5.45 (2.83-10.50) |  | Neutropenia | 5.06 (3.23-7.91) |  | Neutrophil count decreased | 7.83 (4.68-13.10) |
|  |  |  | Pancreatitis acute | 4.99 (1.74-14.33) |  | Pancreatitis acute | 6.56 (1.87-22.98) |
|  |  |  | Myelosuppression | 4.07 (2.06-8.05) |  | Sepsis | 6.05 (2.75-13.31) |
|  |  |  | Pneumocystis jirovecii pneumonia | 3.70 (1.46-9.38) |  | Neutropenia | 4.98 (2.26-10.95) |
|  |  |  | Pneumonia | 2.83 (1.64-4.88) |  | Interstitial lung disease | 2.60 (1.42-4.75) |

ROR, reported odds ratio; 95% CI, 95% confidence interval; PN, peripheral neuropathy; FN, febrile neutropenia; TLS, tumor lysis syndrome.

| Male | |  | Female | |  | Age < 60 | |  | 60 ≤ age | |
| --- | --- | --- | --- | --- | --- | --- | --- | --- | --- | --- |
| Variable | ROR (95% CI) |  | Variable | ROR (95% CI) |  | Variable | ROR (95% CI) |  | Variable | ROR (95% CI) |
| Dermatitis bullous | 64.54 (34.36-121.23) |  | PN | 60.63 (44.70-82.24) |  | Pulmonary toxicity | 155.53 (60.05-402.85) |  | PN | 57.47 (49.68-66.48) |
| PN | 58.63 (50.36-68.25) |  | Skin disorder | 56.62 (28.49-112.55) |  | Metastases to liver | 82.10 (23.29-289.41) |  | Dermatitis bullous | 48.69 (27.46-86.33) |
| Skin disorder | 21.45 (12.92-35.62) |  | Pulmonary toxicity | 51.97 (22.22-121.52) |  | PN | 75.56 (48.08-118.75) |  | Skin disorder | 25.49 (16.55-39.27) |
| Metastases to liver | 19.84 (11.03-35.69) |  | Dermatitis bullous | 42.69 (12.20-149.38) |  | Skin disorder | 33.22 (6.61-166.92) |  | Metastases to liver | 20.00 (11.63-34.40) |
| Pyelonephritis | 18.42 (10.24-33.12) |  | Metastases to liver | 40.63 (15.93-103.61) |  | Malignant neoplasm progression | 30.22 (13.69-66.72) |  | Malignant neoplasm progression | 12.02 (9.32-15.51) |
| Malignant neoplasm progression | 15.03 (11.63-19.42) |  | Malignant neoplasm progression | 15.46 (8.96-26.70) |  | COVID-19 | 16.99 (4.85-59.55) |  | Pyelonephritis | 11.74 (7.20-19.13) |
| Hyperglycaemia | 11.48 (7.74-17.04) |  | Pyelonephritis | 13.38 (5.75-31.13) |  | Myelosuppression | 14.20 (7.06-28.53) |  | Hyperglycaemia | 11.13 (7.61-16.27) |
| Pulmonary toxicity | 7.00 (3.02-16.24) |  | COVID-19 | 11.04 (3.84-31.72) |  | Interstitial lung disease | 6.35 (3.16-12.76) |  | COVID-19 | 6.84 (3.60-12.97) |
| Myelosuppression | 6.94 (5.23-9.20) |  | Myelosuppression | 8.50 (5.01-14.40) |  | Rash | 5.06 (2.30-11.16) |  | Myelosuppression | 6.32 (4.81-8.31) |
| COVID-19 | 5.24 (2.67-10.32) |  | Hyperglycaemia | 6.64 (2.31-19.07) |  | Hyperglycaemia | 7.57 (1.51-37.91) |  | Pulmonary toxicity | 5.72 (2.47-13.25) |
| Pruritus | 4.85 (2.64-8.92) |  | Decreased appetite | 4.83 (2.61-8.94) |  |  |  |  | TEN | 4.63 (3.08-6.97) |
| TEN | 4.11 (2.65-6.37) |  | Erythema multiforme | 4.17 (2.11-8.25) |  |  |  |  | Pruritus | 3.49 (1.84-6.61) |
| Decreased appetite | 2.87 (2.00-4.11) |  | Death | 3.51 (1.38-8.90) |  |  |  |  | SJS | 2.92 (1.90-4.47) |
| SJS | 2.78 (1.79-4.30) |  | Erythema | 3.40 (1.46-7.90) |  |  |  |  | Decreased appetite | 2.74 (2.00-3.74) |
| Rash | 2.27 (1.55-3.34) |  | Drug eruption | 3.29 (1.73-6.28) |  |  |  |  | Drug eruption | 2.62 (1.78-3.85) |
| Erythema | 2.19 (1.15-4.14) |  | Interstitial lung disease | 3.27 (2.09-5.12) |  |  |  |  | Erythema | 2.27 (1.27-4.06) |
| Drug eruption | 2.04 (1.29-3.24) |  | Anaemia | 2.97 (1.65-5.36) |  |  |  |  | Erythema multiforme | 2.25 (1.39-3.66) |
| FN | 1.99 (1.39-2.83) |  | Malaise | 2.93 (1.26-6.81) |  |  |  |  | Rash | 1.94 (1.31-2.87) |
| Diarrhoea | 1.95 (1.30-2.94) |  | Nausea | 2.52 (1.16-5.47) |  |  |  |  | FN | 1.93 (1.37-2.72) |
| Anaemia | 1.85 (1.25-2.73) |  | Neutropenia | 2.32 (1.13-4.79) |  |  |  |  | Diarrhoea | 1.77 (1.20-2.62) |
| Renal impairment | 1.75 (1.20-2.55) |  |  |  |  |  |  |  | Anaemia | 1.74 (1.24-2.44) |
| Interstitial lung disease | 1.72 (1.36-2.18) |  |  |  |  |  |  |  | Renal impairment | 1.66 (1.16-2.38) |
| Pneumonia | 1.51 (1.05-2.18) |  |  |  |  |  |  |  | Interstitial lung disease | 1.58 (1.27-1.96) |

**Supplementary Table S5.** Stratified analysis of ROR and 95% CI of enfortumab vedotin-associated adverse events in JADER.

**Supplementary Table** **S5**. Cont.

| BMI < 18.5 kg/m2 | |  | 18.5 kg/m^2^ ≤ BMI ≤ 24.9 kg/m^2^ | |  | BMI ≤ 25.0 kg/m^2^ | |
| --- | --- | --- | --- | --- | --- | --- | --- |
| Variable | ROR (95% CI) |  | Variable | ROR (95% CI) |  | Variable | ROR (95% CI) |
| Metastases to liver | 138.23  (52.01-367.42) |  | Dermatitis bullous | 111.89  (45.72-273.83) |  | Skin disorder | 80.79 (33.23-196.44) |
| Skin disorder | 77.12 (29.52-201.46) |  | PN | 59.97 (45.84-78.44) |  | PN | 56.81 (35.09-91.97) |
| PN | 48.75 (27.67-85.87) |  | Skin disorder | 33.98 (16.29-70.91) |  | Hyperglycaemia | 15.47 (7.40-32.31) |
| COVID-19 | 47.53 (13.36-169.05) |  | Pulmonary toxicity | 20.12 (9.20-44.03) |  | COVID-19 | 12.99 (2.57-65.62) |
| Pulmonary toxicity | 24.39 (4.82-123.36) |  | COVID-19 | 19.31 (7.55-49.39) |  | Myelosuppression | 9.77 (5.07-18.83) |
| Malignant neoplasm progression | 19.50 (9.32-40.80) |  | Pyelonephritis | 15.63 (7.55-32.37) |  | Pruritus | 8.88 (3.07-25.73) |
| Hyperglycaemia | 15.54 (5.36-45.10) |  | Metastases to liver | 12.91 (3.69-45.18) |  | Malignant neoplasm progression | 7.87 (2.72-22.78) |
| Pyelonephritis | 11.90 (2.37-59.81) |  | Hyperglycaemia | 12.26 (6.62-22.69) |  | TEN | 6.35 (2.20-18.35) |
| Malaise | 5.87 (2.29-15.02) |  | Malignant neoplasm progression | 6.38 (3.35-12.16) |  | Rash | 5.41 (2.81-10.41) |
| Erythema multiforme | 5.00 (1.95-12.79) |  | TEN | 5.43 (2.94-10.03) |  | Erythema | 4.27 (1.48-12.33) |
| Anaemia | 3.98 (1.91-8.31) |  | Pruritus | 4.59 (1.81-11.63) |  | Drug eruption | 3.97 (1.81-8.69) |
| Diarrhoea | 3.35 (1.53-7.36) |  | Decreased appetite | 3.80 (2.38-6.05) |  | FN | 2.96 (1.35-6.49) |
| Myelosuppression | 3.46 (1.20-10.00) |  | Myelosuppression | 3.79 (2.11-6.81) |  | Anaemia | 2.93 (1.25-6.85) |
| Decreased appetite | 3.00 (1.28-7.03) |  | Erythema | 2.88 (1.32-6.24) |  |  |  |
|  |  |  | SJS | 2.66 (1.40-5.07) |  |  |  |
|  |  |  | Anaemia | 2.08 (1.19-3.65) |  |  |  |
|  |  |  | FN | 1.84 (1.07-3.16) |  |  |  |

ROR, reported odds ratio; 95% CI, 95% confidence interval; PN, peripheral neuropathy; COVID-19, Coronavirus disease 2019; TEN, toxic epidermal necrolysis; SJS, Stevens-Johnson syndrome; FN, febrile neutropenia.

**Supplementary Table S6.** Proportion of post-event outcomes of polatuzumab vedotin-associated adverse events.

| **Adverse effects** | **Case (n)** | **Post-events outcome** | | | | | |
| --- | --- | --- | --- | --- | --- | --- | --- |
|  |  | **Recovered** | **Remission** | **Not recovered** | **With sequelae** | **Death** | **Unclear** |
| A) Hematological related AEs |  |  |  |  |  |  |  |
| Lymphocyte count decreased | 116 | 55 (47.4%) | 16 (13.8%) | 32 (27.6%) | 0 (0%) | 0 (0%) | 13 (11.2%) |
| Cytopenia | 38 | 13 (34.2%) | 13 (34.2%) | 7 (18.4%) | 0 (0%) | 0 (0%) | 5 (13.2%) |
| Neutrophil count decreased | 348 | 251 (72.1%) | 64 (18.4%) | 18 (5.2%) | 0 (0%) | 0 (0%) | 15 (4.3%) |
| Platelet count decreased | 203 | 67 (33.0%) | 57 (28.1%) | 61 (30.0%) | 0 (0%) | 2 (1.0%) | 16 (7.9%) |
| White blood cell count decreased | 157 | 102 (65.0%) | 23 (14.7%) | 20 (12.7%) | 0 (0%) | 1 (0.6%) | 11 (7.0%) |
| Anaemia | 113 | 39 (34.5%) | 39 (34.5%) | 35 (30.1%) | 0 (0%) | 0 (0%) | 0 (0%) |
| FN | 106 | 74 (69.8%) | 21 (19.8%) | 6 (5.7%) | 0 (0%) | 1 (0.9%) | 4 (3.8%) |
| Myelosuppression | 51 | 29 (56.9%) | 6 (11.8%) | 9 (17.6%) | 0 (0%) | 1 (2.0%) | 6 (11.8%) |
| Neutropenia | 75 | 52 (69.3%) | 11 (14.7%) | 8 (10.7%) | 0 (0%) | 0 (0%) | 4 (5.3%) |
| Thrombocytopenia | 25 | 13 (52.0%) | 5 (20.0%) | 6 (24.0%) | 0 (0%) | 0 (0%) | 1 (4.0%) |
| B) Infections |  |  |  |  |  |  |  |
| CMV infection reactivation | 29 | 10 (34.5%) | 10 (34.5%) | 7 (24.1%) | 0 (0%) | 0 (0%) | 2 (6.9%) |
| CMV infection | 87 | 46 (52.9%) | 26 (29.9%) | 7 (8.0%) | 0 (0%) | 2 (2.3%) | 6 (6.9%) |
| CMV enterocolitis | 24 | 10 (41.7%) | 7 (29.2%) | 3 (12.5%) | 0 (0%) | 3 (12.5%) | 1 (4.2%) |
| CMV viraemia | 27 | 16 (59.3%) | 7 (25.9%) | 2 (7.4%) | 0 (0%) | 1 (3.7%) | 1 (3.7%) |
| Pneumonia cytomegaloviral | 10 | 4 (40.0%) | 5 (50.0%) | 0 (0%) | 0 (0%) | 1 (10.0%) | 0 (0%) |
| COVID-19 | 15 | 4 (26.7%) | 1 (6.7%) | 4 (26.7%) | 0 (0%) | 2 (13.3%) | 4 (26.7%) |
| Bacteraemia | 10 | 6 (60.0%) | 2 (20.0%) | 1 (10.0%) | 0 (0%) | 1 (10.0%) | 0 (0%) |
| Infection | 11 | 4 (36.4%) | 2 (18.2%) | 2 (18.2%) | 0 (0%) | 3 (27.3%) | 0 (0%) |
| Sepsis | 21 | 9 (42.9%) | 6 (28.6%) | 0 (0%) | 0 (0%) | 5 (23.8%) | 1 (4.8%) |
| Pneumocystis jirovecii pneumonia | 15 | 10 (66.7%) | 3 (20.0%) | 0 (0%) | 0 (0%) | 2 (13.3%) | 0 (0%) |
| Pneumonia | 41 | 22 (53.7%) | 7 (17.1%) | 3 (7.3%) | 0 (0%) | 7 (17.1%) | 2 (4.9%) |
| D) Neurologic related AEs |  |  |  |  |  |  |  |
| CRS | 13 | 9 (69.2%) | 0 (0%) | 1 (7.7%) | 0 (0%) | 0 (0%) | 3 (23.1%) |
| PN | 16 | 5 (31.3%) | 5 (31.3%) | 6 (37.5%) | 0 (0%) | 0 (0%) | 0 (0%) |
| F) Other |  |  |  |  |  |  |  |
| TLS | 30 | 16 (53.3%) | 7 (23.3%) | 2 (6.7%) | 0 (0%) | 2 (6.7%) | 3 (10.0%) |
| Decreased appetite | 25 | 10 (40.0%) | 3 (12.0%) | 10 (40.0%) | 0 (0%) | 0 (0%) | 2 (8.0%) |

AE, adverse event; FN, febrile neutropenia; CMV, cytomegalovirus; COVID-19, Coronavirus disease 2019; CRS, cytokine release syndrome; PN, peripheral neuropathy; TLS, tumor lysis syndrome.

**Supplementary Table S7.** Proportion of post-event outcomes of brentuximab vedotin-associated adverse events.

| **Adverse effects** | **Case (n)** | **Post-events outcome** | | | | | |
| --- | --- | --- | --- | --- | --- | --- | --- |
|  |  | **Recovered** | **Remission** | **Not recovered** | **With sequelae** | **Death** | **Unclear** |
| A) Hematological related AEs |  |  |  |  |  |  |  |
| Neutropenia | 194 | 124 (63.9%) | 29 (14.9%) | 13 (6.7%) | 0 (0%) | 0 (0%) | 28 (14.4%) |
| FN | 188 | 124 (66.0%) | 27 (14.3%) | 9 (4.8%) | 1 (0.5%) | 6 (3.2%) | 21 (11.2%) |
| Neutrophil count decreased | 202 | 128 (63.4%) | 32 (15.8%) | 13 (6.4%) | 0 (0%) | 4 (2.0%) | 25 (12.4%) |
| Myelosuppression | 47 | 22 (68.1%) | 1 (2.1%) | 0 (0%) | 0 (0%) | 0 (0%) | 14 (29.8%) |
| B) Infections |  |  |  |  |  |  |  |
| Pneumocystis jirovecii pneumonia | 28 | 15 (53.6%) | 6 (21.4%) | 3 (10.7%) | 0 (0%) | 4 (14.3%) | 3 (10.7%) |
| Pneumonia | 67 | 25 (37.3%) | 19 (28.4%) | 5 (7.5%) | 0 (0%) | 9 (13.3%) | 9 (13.3%) |
| Sepsis | 29 | 10 (34.4%) | 2 (6.9%) | 0 (0%) | 0 (0%) | 13 (44.8%) | 4 (13.8%) |
| C) Tumor related AEs |  |  |  |  |  |  |  |
| Anaplastic large-cell lymphoma | 47 | 0 (0%) | 0 (0%) | 2 (4.3%) | 0 (0%) | 34 (72.3%) | 11 (23.4%) |
| Hodgkin's disease recurrent | 20 | 0 (0%) | 2 (10.0%) | 4 (20.0%) | 0 (0%) | 6 (30.0%) | 8 (40.0%) |
| Hodgkin's disease | 90 | 4 (4.4%) | 4 (4.4%) | 9 (10.0%) | 0 (0%) | 31 (34.4%) | 42 (46.7%) |
| Peripheral T-cell lymphoma unspecified | 30 | 0 (0%) | 0 (0%) | 1 (3.3%) | 0 (0%) | 19 (63.4%) | 10 (33.3%) |
| Disease progression | 138 | 2 (1.4%) | 6 (4.3%) | 15 (10.9%) | 0 (0%) | 46 (33.3%) | 69 (50.0%) |
| Adult T-cell lymphoma/leukaemia | 12 | 0 (0%) | 0 (0%) | 1 (8.3%) | 0 (0%) | 9 (75.0%) | 2 (16.7%) |
| Condition aggravated | 52 | 5 (9.6%) | 1 (1.9%) | 9 (17.3%) | 0 (0%) | 24 (46.2%) | 13 (25.0%) |
| D) Neurologic related AEs |  |  |  |  |  |  |  |
| PN | 403 | 62 (15.4%) | 90 (22.3%) | 158 (39.2%) | 2 (0.5%) | 0 (0%) | 91 (22.6%) |
| Peripheral sensory neuropathy | 10 | 1 (10.0%) | 3 (30.0%) | 4 (40.0%) | 1 (10.0%) | 0 (0%) | 1 (10.0%) |
| F) Other |  |  |  |  |  |  |  |
| Ileus paralytic | 11 | 5 (45.6%) | 4 (36.4%) | 0 (0%) | 0 (0%) | 1 (9.1%) | 1 (9.1%) |
| TLS | 10 | 3 (30.0%) | 2 (20.0%) | 0 (0%) | 0 (0%) | 3 (30.0%) | 2 (20.0%) |
| Pancreatitis acute | 10 | 2 (20.0%) | 5 (50.0%) | 1 (!0.0%) | 0 (0%) | 1 (10.0%) | 1 (10.0%) |

AE, adverse event; FN, febrile neutropenia; PN, peripheral neuropathy; TLS, tumor lysis syndrome.

| **Adverse effects** | **Case (n)** | **Post-events outcome** | | | | | |
| --- | --- | --- | --- | --- | --- | --- | --- |
|  |  | **Recovered** | **Remission** | **Not recovered** | **With sequelae** | **Death** | **Unclear** |
| A) Hematological related AEs |  |  |  |  |  |  |  |
| Myelosuppression | 68 | 30 (44.1%) | 12 (17.6%) | 6 (8.8%) | 0 (0%) | 7 (10.3%) | 13 (19.1%) |
| FN | 36 | 28 (77.7%) | 2 (5.6%) | 2 (5.6%) | 0 (0%) | 4 (11.1%) | 0 (0%) |
| Anaemia | 37 | 15 (40.5%) | 10 (27.0%) | 8 (21.6%) | 0 (0%) | 0 (0%) | 4 (10.8%) |
| B) Infections |  |  |  |  |  |  |  |
| Pyelonephritis | 16 | 13 (81.3%) | 2 (12.5%) | 0 (0%) | 0 (0%) | 0 (0%) | 1 (6.3%) |
| COVID-19 | 11 | 9 (81.8%) | 1 (9.1%) | 1 (9.1%) | 0 (0%) | 0 (0%) | 0 (0%) |
| Pneumonia | 36 | 17 (47.2%) | 7 (19.4%) | 4 (11.1%) | 1 (2.8%) | 2 (5.6%) | 5 (13.9%) |
| C) Tumor related AEs |  |  |  |  |  |  |  |
| Metastases to liver | 15 | 0 (0%) | 0 (0%) | 6 (40.0%) | 0 (0%) | 2 (13.3%) | 7 (46.7%) |
| Malignant neoplasm progression | 77 | 0 (0%) | 0 (0%) | 12 (15.6%) | 0 (0%) | 28 (36.4%) | 37 (48.1%) |
| D) Neurologic related AEs |  |  |  |  |  |  |  |
| PN | 264 | 25 (9.5%) | 56 (21.2%) | 119 (45.1%) | 5 (1.9%) | 0 (0%) | 59 (22.3%) |
| E) Skin related AEs |  |  |  |  |  |  |  |
| Dermatitis bullous | 12 | 5 (41.7%) | 2 (16.7%) | 4 (33.3%) | 0 (0%) | 0 (0%) | 1 (8.3%) |
| Skin disorder | 23 | 7 (30.4%) | 9 (39.1%) | 2 (8.7%) | 0 (0%) | 3 (13.0%) | 2 (8.7%) |
| TEN | 23 | 5 (21.7%) | 3 (13.0%) | 2 (8.7%) | 0 (0%) | 10 (43.5%) | 3 (13.0%) |
| Pruritus | 11 | 3 (27.3%) | 5 (45.5%) | 2 (18.2%) | 0 (0%) | 0 (0%) | 1 (9.1%) |
| SJS | 23 | 10 (43.5%) | 5 (21.7%) | 1 (4.3%) | 0 (0%) | 2 (8.7%) | 5 (21.7%) |
| Rash | 32 | 16 (50.0%) | 12 (37.5%) | 2 (6.3%) | 0 (0%) | 0 (0%) | 2 (6.3%) |
| Drug eruption | 27 | 15 (55.6%) | 7 (25.9%) | 0 (0%) | 0 (0%) | 0 (0%) | 5 (18.5%) |
| Erythema | 14 | 10 (71.4%) | 3 (21.4%) | 0 (0%) | 0 (0%) | 0 (0%) | 1 (7.2%) |
| Erythema multiforme | 17 | 6 (35.3%) | 7 (41.1%) | 1 (5.9%) | 0 (0%) | 0 (0%) | 3 (17.6%) |
| F) Other |  |  |  |  |  |  |  |
| Hyperglycaemia | 30 | 8 (26.7%) | 8 (26.7%) | 7 (23.3%) | 1 (3.3%) | 3 (10.0%) | 3 (10.0%) |
| Pulmonary toxicity | 10 | 4 (40.0%) | 4 (40.0%) | 1 (10.0%) | 1 (10.0%) | 0 (0%) | 0 (0%) |
| Decreased appetite | 42 | 22 (52.4%) | 12 (28.6%) | 4 (9.5%) | 0 (0%) | 1 (2.4%) | 3 (9.5%) |
| Interstitial lung disease | 96 | 34 (35.4%) | 23 (24.0%) | 15 (15.6%) | 1 (1.0%) | 10 (10.4%) | 13 (13.5%) |
| Diarrhoea | 28 | 15 (53.6%) | 8 (28.6%) | 2 (7.1%) | 0 (0%) | 1 (3.6%) | 2 (7.1%) |
| Renal impairment | 32 | 14 (43.8%) | 6 (18.8%) | 7 (21.9%) | 0 (0%) | 0 (0%) | 5 (15.6%) |

**Supplementary Table S8.** Proportion of post-event outcomes of enfortumab vedotin-associated adverse events.

AE, adverse event; FN, febrile neutropenia; COVID-19, Coronavirus disease 2019; PN, peripheral neuropathy; TLS, tumor lysis syndrome; TEN, toxic epidermal necrolysis; SJS, Stevens-Johnson syndrome.

**Supplementary Table S9.** Weibull parameter of polatuzumab vedotin-associated adverse events.

| **Adverse effects** | **Case (n)** | Scale parameter | Shape parameter |
| --- | --- | --- | --- |
|  |  | α(95%CI) | β(95%CI) |
| A) Hematological related AEs |  |  |  |
| Lymphocyte count decreased | 102 | 9.61 (7.12-12.85) | 0.71 (0.61-0.80) |
| Cytopenia | 14 | 18.76 (7.18-46.01) | 0.64 (0.41-0.94) |
| Neutrophil count decreased | 306 | 34.13 (30.10-38.61) | 0.95 (0.87-1.04) |
| Platelet count decreased | 172 | 32.86 (27.38-39.25) | 0.88 (0.77-0.99) |
| White blood cell count decreased | 136 | 28.84 (23.38-35.36) | 0.87 (0.76-0.98) |
| Anaemia | 108 | 25.21 (19.12-32.97) | 0.74 (0.63-0.85) |
| FN | 83 | 48.11 (38.66-59.43) | 1.07 (0.89-1.26) |
| Myelosuppression | 33 | 30.36 (18.89-47.70) | 0.80 (0.61-1.02) |
| Neutropenia | 57 | 30.99 (22.51-42.09) | 0.89 (0.72-1.08) |
| Thrombocytopenia | 20 | 31.44 (19.68-48.98) | 1.07 (0.72-1.49) |
| B) Infections |  |  |  |
| CMV infection reactivation | 28 | 54.37 (42.53-68.68) | 1.76 (1.27-2.32) |
| CMV infection | 54 | 62.71 (50.94-76.66) | 1.39 (1.10-1.72) |
| CMV enterocolitis | 21 | 112.43 (83.06-149.63) | 1.61 (1.11-2.19) |
| CMV viraemia | 25 | 55.72 (37.24-81.58) | 1.10 (0.78-1.48) |
| Pneumonia cytomegaloviral | 9 | 96.87 (56.34-161.16) | 1.49 (0.81-2.38) |
| COVID-19 | 3 | 73.73 (2.28- 2321.85) | 0.54 (0.16-1.28) |
| Bacteraemia | 8 | 40.16 (19.67-78.17) | 1.23 (0.64-2.06) |
| Infection | 7 | 50.18 (18.75-126.40) | 0.95 (0.49-1.56) |
| Sepsis | 19 | 72.78 (47.11-109.53) | 1.19 (0.80-1.67) |
| Pneumocystis jirovecii pneumonia | 13 | 77.70 (52.14-113.02) | 1.61 (1.00-2.36) |
| Pneumonia | 33 | 65.89 (47.37-90.17) | 1.15 (0.87-1.49) |
| D) Neurologic related AEs |  |  |  |
| CRS | 5 | 87.22 (33.01-220.55) | 1.21 (0.53-2.18) |
| PN | 14 | 68.06 (47.93-94.42) | 1.76 (1.11-2.58) |
| F) Other |  |  |  |
| TLS | 20 | 2.33 (1.63-3.25) | 1.41 (0.99-1.88) |
| Decreased appetite | 28 | 37.28 (18.25-72.81) | 0.75 (0.50-1.07) |

AE, adverse event; FN, febrile neutropenia; CMV, cytomegalovirus; COVID-19, Coronavirus disease 2019; CRS, cytokine release syndrome; PN, peripheral neuropathy; TLS, tumor lysis syndrome.

**Supplementary Table S10.** Weibull parameter of brentuximab vedotin-associated adverse events.

| **Adverse effects** | **Case (n)** | Scale parameter | Shape parameter |
| --- | --- | --- | --- |
|  |  | α(95%CI) | β(95%CI) |
| A) Hematological related AEs |  |  |  |
| Neutropenia | 62 | 3.48 (3.13-3.82) | 1.29 (1.20-1.54) |
| FN | 39 | 20.89 (16.02-26.93) | 1.31 (1.02-1.63) |
| Neutrophil count decreased | 51 | 23.04 (17.99-29.26) | 1.22 (0.99-1.48) |
| Myelosuppression | 3 | 6.55 (3.75-11.37) | 3.17 (1.01-6.88) |
| B) Infections |  |  |  |
| Pneumocystis jirovecii pneumonia | 9 | 77.69 (42.24-137.37) | 1.32 (0.74-2.07) |
| Pneumonia | 14 | 86.91 (37.63-190.70) | 0.73 (0.47-1.04) |
| Sepsis | 8 | 56.33 (13.19-222.24) | 0.58 (0.33-0.88) |
| C) Tumor related AEs |  |  |  |
| Anaplastic large-cell lymphoma | 13 | 24.69 (10.23-56.38) | 0.73 (0.46-1.06) |
| Hodgkin's disease recurrent | 5 | 128.19 (26.41-573.98) | 0.76 (0.32-1.45) |
| Hodgkin's disease | 13 | 144.92 (79.90-254.06) | 1.07 (0.68-1.53) |
| Peripheral T-cell lymphoma unspecified | 1 | - | - |
| Disease progression | 11 | 133.25 (49.16-339.94) | 0.72 (0.41-1.11) |
| Adult T-cell lymphoma/leukaemia | 0 | - | - |
| Condition aggravated | 14 | 28.19 (11.85-63.75) | 0.71 (0.45-1.01) |
| D) Neurologic related AEs |  |  |  |
| PN | 16 | 92.82 (55.10-151.51) | 1.10 (0.71-1.59) |
| Peripheral sensory neuropathy | 7 | 102.35 (46.01-216.65) | 1.18 (0.60-1.98) |
| F) Other |  |  |  |
| Ileus paralytic | 3 | 79.26 (23.33-266.27) | 1.49 (0.45-3.39) |
| TLS | 4 | 4.36 (1.15-15.64) | 1.07 (0.40-2.16) |
| Pancreatitis acute | 3 | 46.71 (16.53-130.61) | 1.71 (0.54-3.77) |

AE, adverse event; FN, febrile neutropenia; PN, peripheral neuropathy; TLS, tumor lysis syndrome.

**Supplementary Table S11.** Weibull parameter of enfortumab vedotin-associated adverse events.

| **Adverse effects** | **Case (n)** | Scale parameter | Shape parameter |
| --- | --- | --- | --- |
|  |  | α(95%CI) | β(95%CI) |
| A) Hematological related AEs |  |  |  |
| Myelosuppression | 18 | 37.05 (18.72-70.62) | 0.78 (0.54-1.05) |
| FN | 23 | 27.00 (18.92-38.07) | 1.28 (0.96-1.62) |
| Anaemia | 20 | 49.39 (29.75-79.58) | 0.99 (0.68-1.38) |
| B) Infections |  |  |  |
| Pyelonephritis | 6 | 50.05 (18.99-126.64) | 1.09 (0.50-1.96) |
| COVID-19 | 3 | 245.75 (158.80-378.55) | 4.06 (1.29-8.86) |
| Pneumonia | 18 | 69.06 (49.16-95.18) | 1.57 (1.04-2.22) |
| C) Tumor related AEs |  |  |  |
| Metastases to liver | 4 | 145.77 (64.25-320.86) | 1.67 (0.67-3.18) |
| Malignant neoplasm progression | 9 | 82.86 (51.75-128.71) | 1.75 (0.92-2.96) |
| D) Neurologic related AEs |  |  |  |
| PN | 71 | 95.48 (77.63-116.64) | 1.21 (0.99-1.46) |
| E) Skin related AEs |  |  |  |
| Dermatitis bullous | 9 | 48.07 (24.40-90.43) | 1.18 (0.67-1.87) |
| Skin disorder | 8 | 26.33 (10.35-62.91) | 0.94 (0.49-1.59) |
| TEN | 12 | 34.73 (19.15-60.76) | 1.12 (0.70-1.63) |
| Pruritus | 6 | 10.55 (7.09-15.33) | 2.64 (1.25-4.65) |
| SJS | 13 | 35.33 (22.56-53.70) | 1.44 (0.90-2.11) |
| Rash | 25 | 13.74 (10.76-17.36) | 1.80 (1.33-2.30) |
| Drug eruption | 16 | 17.76 (9.95-30.73) | 0.97 (0.65-1.35) |
| Erythema | 8 | 26.32 (13.35-49.77) | 1.26 (0.69-1.98) |
| Erythema multiforme | 9 | 24.08 (9.24-59.59) | 0.82 (0.48-1.21) |
| F) Other |  |  |  |
| Hyperglycaemia | 18 | 15.50 (10.15-23.22) | 1.24 (0.83-1.71) |
| Pulmonary toxicity | 1 | - | - |
| Decreased appetite | 22 | 33.51 (21.15-51.87) | 1.03 (0.74-1.35) |
| Interstitial lung disease | 47 | 75.43 (63.03-89.60) | 1.76 (1.39-2.16) |
| Diarrhoea | 18 | 3.42 (2.97-3.84) | 1.19 (0.83-1.60) |
| Renal impairment | 14 | 29.12 (13.79-58.82) | 0.82 (0.52-1.18) |

AE, adverse event; FN, febrile neutropenia; COVID-19, Coronavirus disease 2019; PN, peripheral neuropathy; TLS, tumor lysis syndrome; TEN, toxic epidermal necrolysis; SJS, Stevens-Johnson syndrome.
